# Alternative Oxidase Pathway Optimizes Photosynthesis During Osmotic and Temperature Stress by Regulating Cellular ROS, Malate Valve and Antioxidative Systems

**DOI:** 10.3389/fpls.2016.00068

**Published:** 2016-02-09

**Authors:** Challabathula Dinakar, Abhaypratap Vishwakarma, Agepati S. Raghavendra, Kollipara Padmasree

**Affiliations:** ^1^Department of Plant Sciences, School of Life Sciences, University of HyderabadHyderabad, India; ^2^Department of Life Sciences, School of Basic and Applied Sciences, Central University of Tamil NaduThiruvarur, India; ^3^Department of Biotechnology and Bioinformatics, School of Life Sciences, University of HyderabadHyderabad, India

**Keywords:** alternative oxidase, cytochrome oxidase, photosynthesis, reactive oxygen species, respiration, salicylhydroxamic acid

## Abstract

The present study reveals the importance of alternative oxidase (AOX) pathway in optimizing photosynthesis under osmotic and temperature stress conditions in the mesophyll protoplasts of *Pisum sativum*. The responses of photosynthesis and respiration were monitored at saturating light intensity of 1000 μmoles m^–2^ s^–1^ at 25°C under a range of sorbitol concentrations from 0.4 to 1.0 M to induce hyper-osmotic stress and by varying the temperature of the thermo-jacketed pre-incubation chamber from 25 to 10°C to impose sub-optimal temperature stress. Compared to controls (0.4 M sorbitol and 25°C), the mesophyll protoplasts showed remarkable decrease in NaHCO_3_-dependent O_2_ evolution (indicator of photosynthetic carbon assimilation), under both hyper-osmotic (1.0 M sorbitol) and sub-optimal temperature stress conditions (10°C), while the decrease in rates of respiratory O_2_ uptake were marginal. The capacity of AOX pathway increased significantly in parallel to increase in intracellular pyruvate and reactive oxygen species (ROS) levels under both hyper-osmotic stress and sub-optimal temperature stress under the background of saturating light. The ratio of redox couple (Malate/OAA) related to malate valve increased in contrast to the ratio of redox couple (GSH/GSSG) related to antioxidative system during hyper-osmotic stress. Further, the ratio of GSH/GSSG decreased in the presence of sub-optimal temperature, while the ratio of Malate/OAA showed no visible changes. Also, the redox ratios of pyridine nucleotides increased under hyper-osmotic (NADH/NAD) and sub-optimal temperature (NADPH/NADP) stresses, respectively. However, upon restriction of AOX pathway by using salicylhydroxamic acid (SHAM), the observed changes in NaHCO_3_-dependent O_2_ evolution, cellular ROS, redox ratios of Malate/OAA, NAD(P)H/NAD(P) and GSH/GSSG were further aggravated under stress conditions with concomitant modulations in NADP-MDH and antioxidant enzymes. Taken together, the results indicated the importance of AOX pathway in optimizing photosynthesis under both hyper-osmotic stress and sub-optimal temperatures. Regulation of ROS through redox couples related to malate valve and antioxidant system by AOX pathway to optimize photosynthesis under these stresses are discussed.

## Introduction

The mitochondrial oxidative electron transport chain in higher plants is branched at ubiquinone, leading to cyanide sensitive cytochrome oxidase (COX) and cyanide resistant alternative oxidase (AOX) pathways (Millar et al., 2011). The COX pathway transfers electrons from ubiquinone to molecular O_2_ through complex III and complex IV and generates a proton gradient which is coupled to ATP synthesis. The electron transport through AOX pathway is mediated by a quinol oxidase and uncoupled from ATP synthesis. However, energy is liberated as heat when the AOX pathway is operative (Day and Wiskich, 1995; Siedow and Umbach, 2000; Schertl and Braun, 2014; Pu et al., 2015). Although, AOX catalyzes the energy-wasteful respiration, its (up) regulation in terms of activity, engagement and expression during development and biotic/abiotic stresses indicates its physiological importance other than thermogenesis (Fung et al., 2006; Matos et al., 2007; Giraud et al., 2008; Arnholdt-Schmitt, 2009; Vanlerberghe et al., 2009; Fu et al., 2010; Florez-Sarasa et al., 2011; Cvetkovska and Vanlerberghe, 2013; Vishwakarma et al., 2014; Garmash et al., 2015; Rogov and Zvyagilskaya, 2015).

Mitochondrial functions contribute to the metabolic flexibility that is essential for plant cells to adjust to highly variable environment (Vanlerberghe, 2013). The functioning of AOX pathway through hand-in-hand cooperation with COX pathway to optimize photosynthetic metabolism (Padmasree and Raghavendra, 1999a,b,c, 2001a,b; Yoshida et al., 2006; Feng et al., 2007; Strodtkötter et al., 2009; Dinakar et al., 2010a,b; Florez-Sarasa et al., 2011; Bailleul et al., 2015; Vishwakarma et al., 2015) and its active participation in balancing carbon/nitrogen availability with sink capacity or antioxidant defense system has added new dimensions to its existence in leaf cells (Parsons et al., 1999; Vanlerberghe et al., 2002; Sieger et al., 2005; Umbach et al., 2005; Yoshida et al., 2007; Gandin et al., 2009, 2014; Dahal et al., 2014). Thus the relative contribution of COX and AOX pathways to total respiration is known to be flexible and dependant on environmental conditions (Gonzalez-Meler et al., 1999; Searle et al., 2011; Liu et al., 2015).

Water stress affects various parameters including stomatal conductance, root growth, leaf number, total leaf area, photosynthetic quantum yield, ATP, NADPH synthesis and the utilization of assimilates (Vandoorne et al., 2012; Cano et al., 2014; Esmaeilpour et al., 2015) along with the partitioning of electrons between the COX and AOX pathways (Ribas-Carbo et al., 2005). There is a considerable ambiguity in the partitioning of electrons between these pathways. In soyabean and wheat, water stress caused a significant shift of electrons from the COX to the AOX pathway while in leaves of bean and pepper water stress decreased SHAM-resistant respiration, with no effect on cyanide-resistant respiration (Gonzalez-Meler et al., 1997; Ribas-Carbo et al., 2005; Vassileva et al., 2009). Several other studies suggested that changes in electron partitioning between the two respiratory pathways under a given stress were mostly due to the decrease in the activity of the COX pathway rather than an increase in the activity of the AOX pathway (Peñeulas et al., 1996; Lambers et al., 2005; Galle et al., 2010). An increase in the expression of AOX genes and its activity in photosynthetic tissues has been reported in plants subjected to low/high temperatures (Vanlerberghe and McIntosh, 1992a,b; Fiorani et al., 2005; Wang et al., 2011) or water stress (Bartoli et al., 2005; Ribas-Carbo et al., 2005). Several reports proposed that the AOX pathway maintains electron flow during cold conditions to alleviate the cellular reactive oxygen species (ROS; Purvis et al., 1995; Armstrong et al., 2008; Grabelnych et al., 2014). The ability of AOX pathway to maintain flux in the cold was suggested to be due to (i) its reduced sensitivity to temperature as compared to COX pathway (Kiener and Bramlage, 1981; McNulty and Cummins, 1987; Stewart et al., 1990b) and (ii) an increase in the *de novo* synthesis of AOX protein (Stewart et al., 1990a,b; Vanlerberghe and McIntosh, 1992a; Gonzalez-Meler et al., 1999; Ribas-Carbo et al., 2000). However, the studies of Kühn et al. (2015) suggested that any decrease in electron flux through the COX or AOX pathways trigger common as well as distinct cellular responses which are in-turn dependent on the growth conditions.

Osmotic and temperature stresses are common abiotic stresses to which plants are frequently exposed under conditions of drought and flooding/frost in natural environment. Long term exposure to any biotic or abiotic stress conditions may cause cellular damage and cell death in susceptible plants. However, during short term exposure, the plants adapt or acclimatize to these stress conditions by various mechanisms. Intracellular adjustments like alteration in redox status, ROS and antioxidant levels, particularly mediated by mitochondria are essential for plant cells to acclimatize with changing environmental conditions to maintain redox homeostasis (Foyer and Noctor, 2003, 2005; Baier and Dietz, 2005; Gechev et al., 2006; Noctor, 2006; Navrot et al., 2007; Noctor et al., 2007; Dinakar et al., 2010a; Scheibe and Dietz, 2012; Tripathy and Oelmüller, 2012; Vishwakarma et al., 2014, 2015; Considine et al., 2015; Deng et al., 2015; Sevilla et al., 2015; Zhao et al., 2015). Also, it is intriguing to know that the same parameters were found to be crucial in mediating the beneficial interactions between chloroplasts and mitochondria to optimize photosynthetic carbon assimilation under optimal light and CO_2_ (Padmasree and Raghavendra, 1999c; Dinakar et al., 2010a; Yoshida et al., 2011). However, it is not clear which pathway (COX or AOX) of mitochondrial electron transport would play a crucial role in optimizing photosynthesis under hyper-osmotic stress or sub-optimal temperature stress. Therefore, the present study was performed using mesophyll protoplasts of pea as the model system to examine the importance of AOX pathway over COX pathway and its coordination with malate valve and glutathione redox system in regulating cellular ROS to optimize photosynthesis under hyper-osmotic and sub-optimal temperature stresses.

## Materials and Methods

### Plant Material and Isolation of Mesophyll Protoplasts

Pea plants (*Pisum sativum* L. cv. Arkel; seeds obtained from Pocha seeds, Pune, India) were grown outdoors under natural photoperiod of approximately 12 h and average daily temperatures of 30°C day/20°C night. The second pair of fully expanded leaves were picked from 8 to 10 days old plants and were used for isolating mesophyll protoplasts. About 10 pairs of leaves were excised from the plants and mesophyll protoplasts were isolated from leaf strips devoid of lower epidermis by enzymatic digestion with 2% (w/v) Cellulase Onozuka R-10 and 0.2% (w/v) Macerozyme R-10 (Yakult Honsha Co. Ltd, Nishinomiya, Japan), under low light intensities of 50–100 μmol m^–2^ s^–1^. The protoplasts were collected by filtration through 60 μm nylon filter and purified by centrifugation at 100 *g* for 5 min, thrice at 4°C. The protoplasts were finally stored on ice in a suspension medium containing 10 mM Hepes-KOH, pH 7.0, 0.4 M sorbitol, 1.0 mM CaCl_2_, and 0.5 mM MgCl_2_ until further use and chlorophyll was estimated (Padmasree and Raghavendra, 1999a). The purity of protoplast preparation normally ranged from 90 to 97%.

### Stress Treatments

Mesophyll protoplasts equivalent to 12 μg Chl were subjected to hyper-osmotic stress or sub-optimal temperature stress under a saturating light intensity (1000 μmol m^–2^ s^–1^) in the pre-incubation chamber by increasing (step wise) the concentration of sorbitol in the pre-incubation medium from 0.4 M (isotonic) to 1.0 M (hypertonic) or by decreasing the temperature in pre-incubation chamber from 25°C to 10°C using a refrigerated circulatory water bath (Julabo F10) for 10 min, respectively (Dinakar et al., 2010b). Protoplast samples pre-incubated at 1000 μmol m^–2^ s^–1^, 25°C temperature and 0.4 M sorbitol were treated as controls (Saradadevi and Raghavendra, 1994). NaHCO_3_ (1.0 mM) is added to the pre-incubation media so as to avoid photorespiration and associated O_2_ burst. The composition of the pre-incubation medium used were same as that of reaction medium (other than sorbitol) described in Dinakar et al. (2010b).

### Monitoring Total Respiration and Photosynthesis

After hyper-osmotic and temperature stress treatments in the presence or absence of SHAM, mesophyll protoplasts equivalent to 10 μg Chl were transferred from pre-incubation chamber to Clark-type oxygen electrode cuvette and the total rates of respiration and photosynthesis (carbon assimilation/PS II activity) were measured polarographically in a reaction medium as described in Dinakar et al. (2010a). In controls, as the rates of respiratory O_2_ uptake and photosynthetic O_2_ evolution attained steady state after 3 min in dark and 4 min after switching on light, respectively, we restricted to monitor respiration and photosynthesis during steady state for 5 and 10 min, in the dark and light, respectively, using a Clark type oxygen electrode system, controlled by Hansa-Tech software at 25°C. Saturating light (1000 μmol m^–2^ s^–1^) was provided by a 35 mm slide projector (with xenophot [halogen] lamp, 24 V/150 W). The photosynthetic carbon assimilation rates were measured as NaHCO_3_ (1.0 mM) dependent O_2_ evolution and PS II activity was measured as *p*-BQ-dependent (1.0 mM) O_2_ evolution in the presence of an uncoupler (5 mM NH_4_Cl). Oxygen content (253 μM) in the electrode chamber was pre-calibrated at 25°C with air saturated water using sodium dithionate.

### Capacity of COX and AOX Pathway

The capacity of AOX pathway was determined as the O_2_ uptake sensitive to 10 mM SHAM in the presence of 1 mM KCN (Vanlerberghe et al., 2002), while the capacity of COX pathway was determined as the O_2_ uptake sensitive to 1 mM KCN in the presence of both 10 mM SHAM and 1 μM carbonyl cyanide m-chlorophenylhydrazone (CCCP, an uncoupler) as adenylates determine the flux of electrons through COX pathway (Dinakar et al., 2010a,b).

### Protein Extraction and Immunodetection

After stress treatments, mesophyll protoplasts equivalent to 10 μg Chl were withdrawn and centrifuged at 100 *g* for 1 min. The pelleted protoplasts were snap frozen in liquid nitrogen and homogenized in 125 mM Tris–HCl (pH 6.8) containing 5% (w/v) SDS and 1 mM PMSF. The homogenate was centrifuged at 10,000 *g* for 10 min. Protein estimation was done according to the method of Lowry et al. (1951). SDS-PAGE of mesophyll protoplast proteins was performed according to Laemmli (1970). The proteins separated on 12.5% SDS-PAGE were transferred electrophoretically from the gel onto polyvinylidene difluoride (PVDF) membranes (Towbin et al., 1979). The blots were probed with 1:100 dilution of D1 antibodies (Agrisera, Vännäs, Sweden) followed by 1:5000 dilution of goat antirabbit IgG alkaline phosphatase conjugate and developed using nitro-blue-tetrazolium chloride and 5-bromo-4-chloro-3-indolyl phosphate as substrates.

### Detection of Reactive Oxygen Species (ROS)

Intracellular production of ROS was measured by using a non polar fluorescent dye 2, 7, -dichlorofluorescein diacetate (H_2_DCF-DA), which is converted to membrane – impermeable polar derivative H_2_DCF by cellular esterases and rapidly oxidized to highly fluorescent DCF by intracellular H_2_O_2_ and other peroxides. Mesophyll protoplasts loaded with 5 μM H_2_DCF-DA (Dinakar et al., 2010a) were subjected to hyper-osmotic and sub-optimal temperature stress for 10 min at saturating light intensities (1000 μmol m^–2^ s^–1^). Immediately, after stress treatments, DCF fluorescence of mesophyll protoplasts was measured by using a Hitachi F- 4010 fluorescence spectrophotometer with excitation and emission wavelengths set at 488 and 525 nm, respectively. DCF fluorescence of protoplasts pre-incubated under a saturating light intensity (1000 μmol m^–2^ s^–1^) at 25°C and 0.4 M sorbitol were treated as controls.

### Quantification of Pyruvate and Adenylates

After stress treatments at saturating light, the metabolic reactions of mesophyll protoplasts were quenched with HClO_4_ as described in Padmasree and Raghavendra (1999a). The samples neutralized with KOH were centrifuged at 7000 *g* and the cleared supernatant was used for estimation of pyruvate, ATP and ADP. The intracellular levels of pyruvate were measured spectrophotometrically using enzymatic assay coupled to NADH oxidation as described in Dinakar et al. (2010b). Similarly, the ATP levels were measured using enzymatic assay coupled to NADPH formation while the ADP levels were measured by coupling to NADH utilization (Padmasree and Raghavendra, 1999a).

### Quantification of Malate and OAA

After exposure to stress treatments in the presence and absence of SHAM, at saturating light, aliquots of mesophyll protoplasts equivalent to 100 μg Chl ml^–1^ were quenched with HClO_4_ and snap frozen in liquid nitrogen. After neutralization, the samples were centrifuged at 100 *g* and the supernatant was used for the estimation of intracellular levels of malate and OAA spectrophotometically. The malate was estimated by incubating the supernatant for 10 min at 25°C in the reaction medium containing 100 mM Tris-HCl, 630 mM hydrazine sulfate, 1.0 mM EDTA pH 9.0, 1.5 mM NAD. The reaction is initiated by the addition of 30U MDH as the concentration of malate is proportional to the amount of NAD reduced at 340 nm (Heineke et al., 1991). Further, the cellular levels of oxaloacetate was calculated from the equation of [(oxoglutarate) × (aspartate)]/[(glutamate) × (6.61)], as suggested by Heineke et al. (1991) based on the equilibrium of glutamate oxaloacetate transaminase (GOT; *K* = 6.61, Veech et al., 1969). The levels of oxoglutarate, aspartate and glutamate were determined as described in Bergmeyer (1983) by enzymatic assays coupled to NAD(H) oxidation or reduction.

### Quantification of GSH and GSSG

After stress treatments in the presence and absence of SHAM, protoplast samples equivalent to 100 μg Chl were withdrawn and mixed immediately with 7% sulfosalicylic acid and snap frozen in liquid nitrogen. The samples were thawed and centrifuged for 10 min. 20 μl of 7.5 M triethanolamine was added to the supernatant to neutralize the samples. Total, oxidized, reduced glutathione was determined spectrophotometrically at 412 nm by the cycling method described by Griffith (1980).

### Quantification of NAD(P) and NAD(P)H

Mesophyll protoplasts equivalent to 25 μg Chl were withdrawn from the pre-incubation chamber with and without SHAM after the stress treatments. The samples were centrifuged at 3000 *g* for 2 min and the pelleted protoplasts were homogenized either in 0.2 N HCl or in 0.2 M NaOH for NAD(P)^+^ and NAD(P)H extraction, respectively. The homogenate was centrifuged at 10,000 *g* for 10 min at 4°C. The supernatant was boiled for 1 min and rapidly cooled on ice. For NAD(P)^+^ measurement the final pH of supernatant was brought between 5.0 and 6.0, while for NAD(P)H measurement the final pH was adjusted between 7.0 and 8.0. Pyridine nucleotides were quantified by monitoring phenazine methosulfate-catalyzed reduction of dichlorophenolindophenol (Queval and Noctor, 2007). For NAD^+^ and NADH assay, the reaction was started by the addition of ethanol in presence of alcohol dehydrogenase. On the other hand, for NADP^+^ and NADPH assay, the reaction was started by addition of Gluocose-6-phosphate dehydrogenase in the presence of Glucose-6-phosphate. The decrease in A_600_ was monitored for 3 min and concentrations of corresponding pyridine nucleotides were calculated using relevant standards (Vishwakarma et al., 2015).

### Assay of NADP-MDH

Mesophyll protoplasts equivalent to 40 μg Chl were withdrawn from the pre-incubation chamber with and without SHAM after the stress treatments. NADP-dependent MDH was extracted and assayed according to Dutilleul et al. (2003). The NADP-MDH was extracted in buffer containing 25 mM Hepes-KOH (pH 7.5), 10 mM MgSO_4_, 1 mM Na_2_EDTA, 5 mM DTT, 1 mM phenylmethylsulfonyl fluoride, 5% (w/v) insoluble polyvinylpyrrolidone, and 0.05% (v/v) Triton X-100. The homogenate was centrifuged for 5 min at 10,000 *g* (4°C). The actual NADP-MDH activity was measured directly from supernatant (2.5 μg chl) in assay buffer. Assay buffer was comprised of 25 mM Tricine-KOH (pH 8.3), 150 mM KCl, 1 mM EDTA, 5 mM DTT, 0.2 mM NADPH, and 2 mM oxaloacetate, plus sample. To fully activate the enzyme, supernatant (2.5 μl chl) was pre-incubated for 30 min at 25°C in 40 mM Tricine-KOH (pH 9.0), 0.4 mM Na_2_EDTA, 120 mM KCl, 100 mM DTT, and 0.0025% (v/v) Triton X-100. After incubation, 2 mM oxaloacetate and 0.2 mM NADPH were added into total reaction volume and activity was measured at 340 nm.

### Assay of Superoxide Dismutase (SOD; E.C. 1.15.1.1), Catalase (CAT; E.C. 1.11.1.6), and Glutathione Reductase (GR; E.C. 1.6.4.2)

Mesophyll protoplasts equivalent to 100 μg Chl in 600 μl were withdrawn from the pre-incubation chamber with and without SHAM after the stress treatments. The samples were centrifuged at 100 *g* for 1 min and the pelleted protoplasts were snap frozen in liquid nitrogen. The samples were homogenized in 50 mM phosphate buffer pH 7.0 containing 1 mM PMSF and centrifuged at 10,000 *g* for 10 min. The supernatant was used for enzymatic assays of superoxide dismutase (SOD), catalase (CAT), and glutathione reductase (GR). The protein concentration in the enzyme extracts were determined by Lowry et al. (1951) using defatted BSA as standard. The SOD activity was determined following the method of Beauchamp and Fridovich (1971). CAT activity was measured spectrophotometrically by following the oxidation of H_2_O_2_ at 240 nm according to the method of Patterson et al. (1984) and GR activity was determined by modifying the method of Jiang and Zhang (2001). Others details were followed as described in Dinakar et al. (2010a).

### Replications

The data presented are the average values of results (+SE) from atleast four repetitions conducted on different days. The differences between treatments were analyzed by one-way ANOVA, Student–Newman–Keuls method of multiple comparison analysis using SigmaStat 3.1 software (San Jose, CA, USA).

## Results

### Photosynthetic Carbon Assimilation and Respiration in Mesophyll Protoplasts Pre-incubated Under Hyper-Osmoticum and Sub-Optimal Temperatures at Saturating Light

**FIGURE 1:**
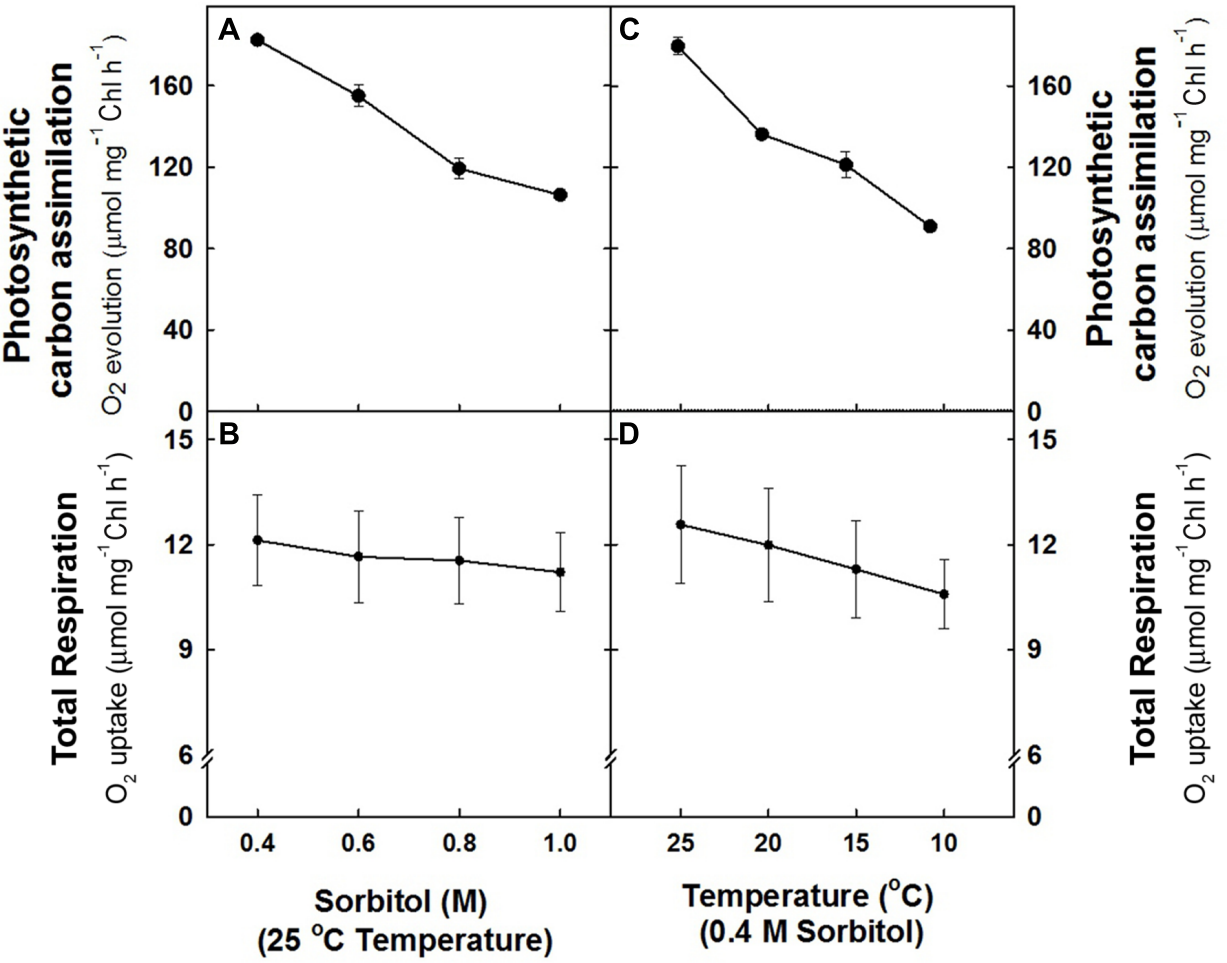
**The rates of photosynthetic O_2_ evolution **(A,C)** and total respiration **(B,D)** in mesophyll protoplasts of pea pre-incubated for 10 min at a saturating light intensity of 1000 μmol m^–2^ s^–1^ under different concentrations of sorbitol (0.4 to 1.0 M) in the reaction media at 25°C and at different temperatures (25 –10°C) at 0.4 M sorbitol in the reaction media.** After exposing the mesophyll protoplasts to different osmotic and temperature treatments in light, the respiratory rates were measured for 5 min in darkness. The photosynthesis rates were measured as NaHCO_3_-dependent (1.0 mM) O_2_ evolution for 10 min in light (1000 μmol m^–2^ s^–1^) using Clark-type oxygen electrode.

In the study, the effect of hyper-osmotic stress and sub-optimal temperature stress on photosynthetic carbon assimilation (NaHCO_3-_-dependent O_2_ evolution) and respiration (O_2_ uptake) was monitored in mesophyll protoplasts under light. The O_2_ evolution rates (182.5 ± 3 μmol mg^–1^ Chl h^–1^) observed at 0.4 M sorbitol (isotonic) at 25°C in light (control) declined remarkably upto ≤42% as the concentration of sorbitol was increased to 1.0 M (**Figure 1A**). In contrast, the rates of O_2_ uptake (12.12 + 1.3 μmol mg^–1^ Chl h^–1^) observed at 0.4 M sorbitol at 25°C temperature (control) decreased marginally by 7% of control with increase in sorbitol concentration to 1.0 M (**Figure 1B**). Similarly, any decrease in temperature of pre-illumination chamber at 0.4 M sorbitol in light also showed a profound effect on rates of O_2_ evolution as compared to rates of O_2_ uptake. Mesophyll protoplasts pre-incubated at 10°C showed a remarkable decrease in O_2_ evolution rates (≤49%) while the decrease in O_2_ uptake rates (≤13%) were marginal as compared to control (**Figures 1C,D**).

### Effects of Hyper-Osmotic Stress and Sub-Optimal Temperature Stress on the Capacity of COX and AOX Pathways

**FIGURE 2:**
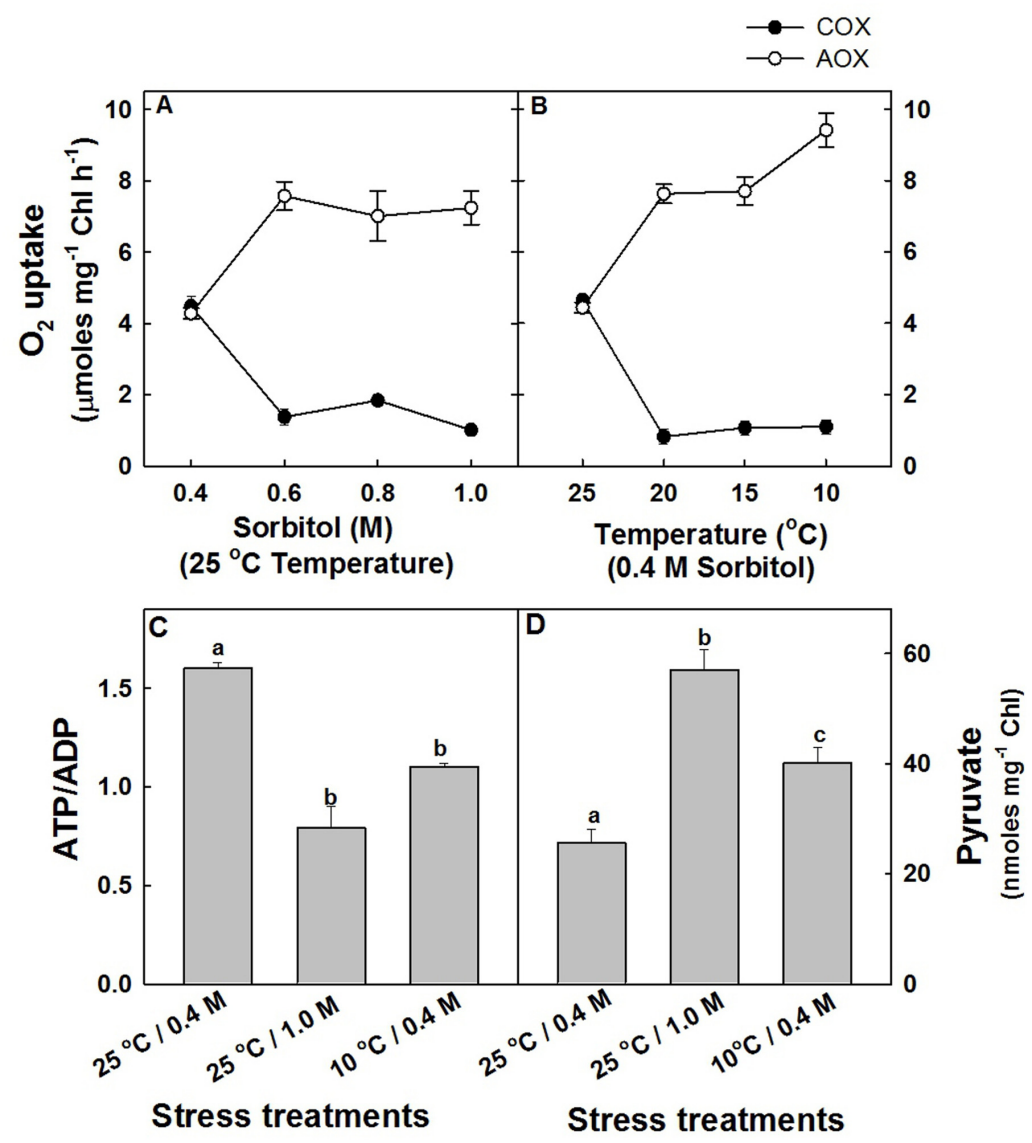
**The capacity of COX and AOX pathways of mitochondrial electron transport chain in mesophyll protoplasts of pea pre-incubated for 10 min at a saturating light intensity of 1000 μmol m^–2^ s^–1^ under different concentrations of sorbitol (0.4 to 1.0 M) in the reaction media at 25°C **(A)** and at different temperatures (25 to 10°C) under 0.4 M sorbitol **(B)**.** The open circles indicate the AOX pathway capacity while the closed circles indicate the COX pathway capacity. The changes in intracellular ATP/ADP **(C)** and pyruvate levels **(D)** in mesophyll protoplasts pre-incubated under 0.4 M (control), 1.0 M sorbitol (osmotic stress) at 25°C and 0.4 M sorbitol at 10°C (temperature stress) respectively, at a saturating light intensity of 1000 μmol m^–2^ s^–1^ for 10 min. Values represent the mean (±SE) of four experiments and different letters represent values that are statistically different (ANOVA test, *p* ≤ 0.05).

Although the effects of hyper-osmotic stress and sub-optimal temperature stress on total respiratory O_2_ uptake of mesophyll protoplasts were marginal, the *in vivo* rates of COX (COX capacity) and AOX (AOX capacity) pathways were modulated significantly. In mesophyll protoplasts which were exposed to increasing sorbitol concentration at 25°C in saturating light, the capacity of COX pathway was decreased drastically by 77%, while the capacity of AOX pathway was stimulated by 70% at 1.0 M sorbitol as compared to protoplasts in 0.4 M sorbitol at 25°C under saturating light (**Figure 2A**). A similar trend was observed in response to sub-optimal temperature stress. With decreasing temperature under 0.4 M sorbitol at saturating light, the capacity of COX pathway of mesophyll protoplasts decreased remarkably by ≤76% and the AOX pathway increased significantly by ≤1.2 fold at 10°C as compared to protoplasts pre-incubated at 25°C under saturating light (**Figure 2B**). Since the decrease in COX capacity and the increase in AOX capacity were maximum at 1.0 M sorbitol and 10°C temperature, in all further experiments, the stress treatments were restricted to 1.0 M sorbitol at 25°C to impose hyper-osmotic stress and at 10°C under 0.4 M sorbitol to impose sub-optimal temperature stress under the background of light.

In light, as most of the cellular demands for ATP are met by COX pathway activity, the changes in adenylates (ATP, ADP, and ATP/ADP) which act as a proof of changes in COX pathway capacity were analyzed (Supplementary Figure S1 and **Figure 2C**). A decrease in ATP/ADP levels at both hyper-osmotic stress (51%) and sub-optimal temperature stress (31%) positively correlated with the decrease in COX pathway capacity (**Figure 2C**). Similarly, the intracellular concentration of pyruvate which is one among the important factors known to stimulate the activity of AOX are increased significantly by 2.22-fold and 56% at hyper-osmotic stress and sub-optimal temperature stress, respectively (**Figure 2D**).

### Effect of Restriction of AOX Pathway on Total Respiration, Photosynthetic Carbon Assimilation and PSII Activities Under Osmotic and Temperature Stress in Light

**FIGURE 3:**
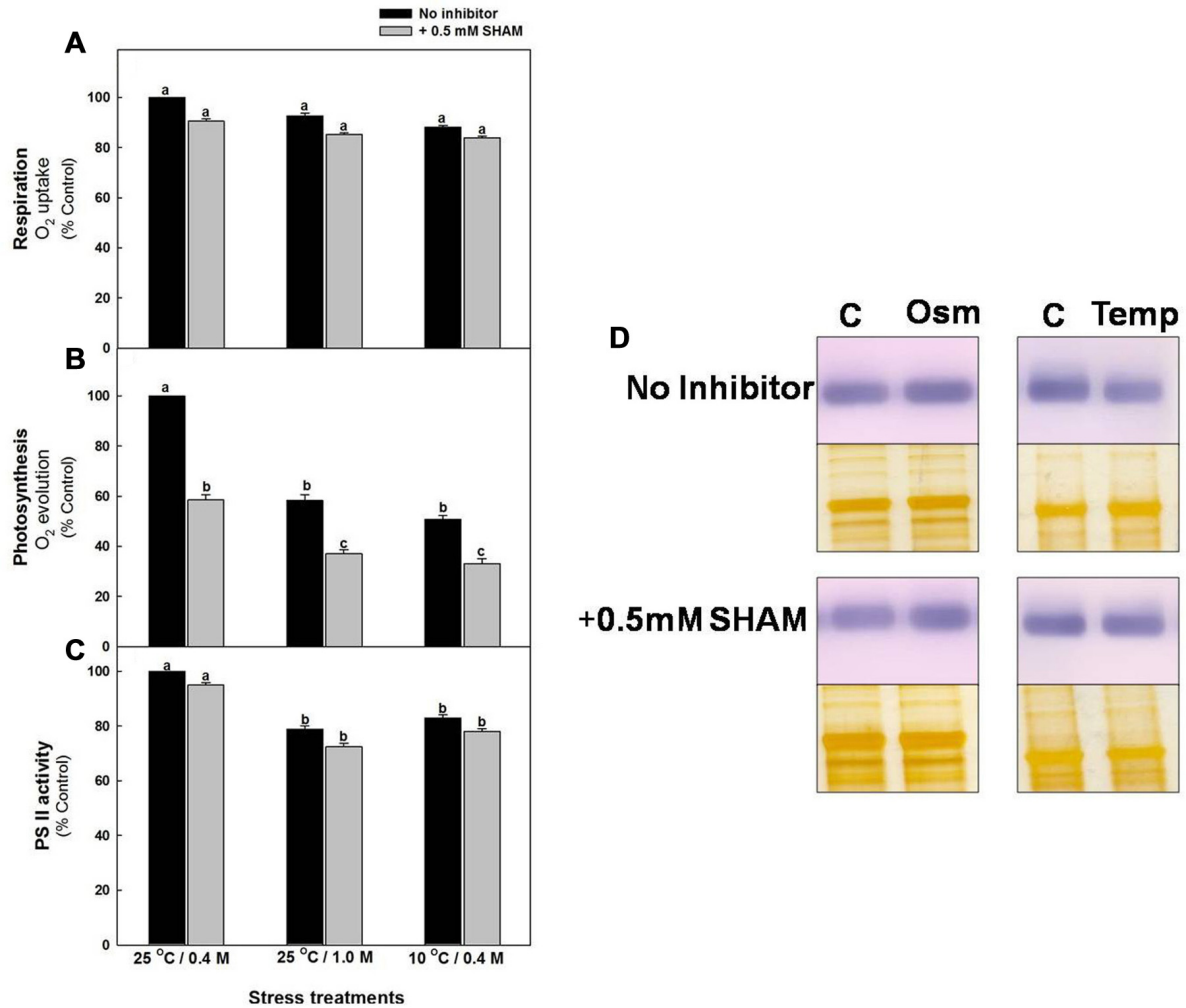
**Effect of 0.5 mM SHAM on respiration **(A)**, photosynthesis **(B)** and PS II activity **(C)** measured in mesophyll protoplasts pre-incubated under control, osmotic and temperature stress conditions with or without 0.5 mM SHAM.** Different letters represent values that are statistically different (ANOVA test, *P* ≤ 0.05). **(D)** Western blot analysis of D1 protein (32 kDa) from mesophyll protoplasts pre-incubated at a saturating light intensity of 1000 μmol m^–2^ s^–1^ under 0.4 M sorbitol (control, **C)** 1.0 M sorbitol (osmotic stress; Osm) and 0.4 M sorbitol at 10°C (temperature stress; Temp) for 10 min in the presence and absence of 0.5 mM SHAM. After the treatments mesophyll protoplasts were homogenized in the extraction buffer and the proteins (8 μg) were separated on SDS-PAGE. Proteins were transferred to PVDF membranes and were probed with the antibodies raised against D1. Equal loading of protein was confirmed by silver staining of a duplicate gel.

The respiratory O_2_ uptake rates of mesophyll protoplasts decreased marginally (≤13%) after pre-incubation under hyper-osmotic stress or sub-optimal temperature stress at saturating light as compared to the rates under 0.4 M sorbitol at 25°C in light (control). Pre-incubation of samples in the presence of SHAM further decreased the rates of respiratory O_2_ uptake up to 14%, both under hyper-osmotic stress and sub-optimal temperature stress, respectively (**Figure 3A**). In contrast to respiration, the NaHCO_3_-dependent photosynthetic O_2_ evolution rates were decreased remarkably by 42% and 49%, respectively, under hyper-osmotic stress and sub-optimal temperature stress when compared with control and the decrease was significantly aggravated to 67% upon addition of SHAM under both osmotic or temperature stress conditions (**Figure 3B**).

Similar to photosynthetic carbon assimilation, PSII activity of mesophyll protoplasts decreased by <21% of control upon exposure to hyper-osmotic stress or sub-optimal temperature stress and the decrease was aggravated up to <28% with addition of SHAM under both osmotic and temperature stress (**Figure 3C**). D1 protein, an important component of PS II showed marginal changes under sub-optimal temperature stress as compared to control, while the changes under 1.0 M sorbitol over-lapping with SHAM were negligible (**Figure 3D**).

### Effect of Restriction of AOX Pathway on Total Cellular ROS Levels and Redox Ratios Under Hyper-Osmotic Stress and Sub-Optimal Temperature Stress in Light

**FIGURE 4:**
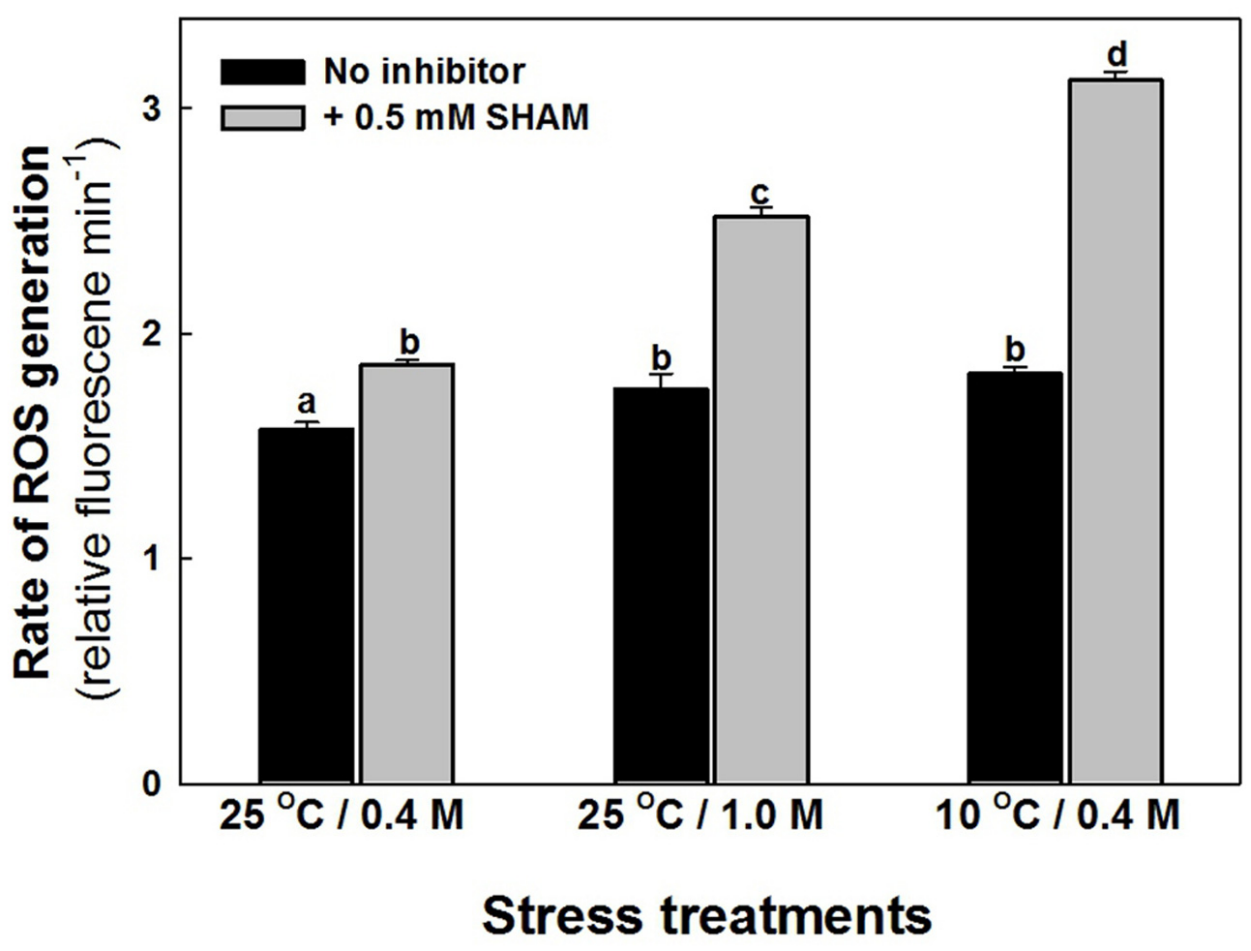
**Effect of 0.5 mM SHAM on intracellular levels of ROS, in mesophyll protoplasts pre-incubated under 0.4 M (control), 1.0 M sorbitol (osmotic stress) at 25°C and 0.4 M sorbitol at 10°C (temperature stress), respectively, at a saturating light intensity of 1000 μmol m^–2^ s^–1^ for 10 min.** ROS levels were measured using ROS-sensitive probe H_2_DCF-DA. DCF fluorescence of the mesophyll protoplasts after incubation for 10 min in stress conditions (1,000 μmol m^–2^ s^–1^) in the absence and presence of SHAM was measured using Hitachi F-4010 fluorescence spectrophotometer with excitation and emission wavelengths set at 488 and 525 nm, respectively. Different letters represent values that are statistically different (ANOVA test, *P* ≤ 0.05).

The intracellular ROS levels of mesophyll protoplasts are increased marginally as compared to control when preincubated under hyper-osmotic stress or sub-optimal temperature stress (**Figure 4**). Parallel to the effect on photosynthesis, the increase in ROS levels were aggravated significantly on super-imposition of SHAM with hyper-osmotic and sub-optimal temperature stresses (**Figure 4**).

**FIGURE 5:**
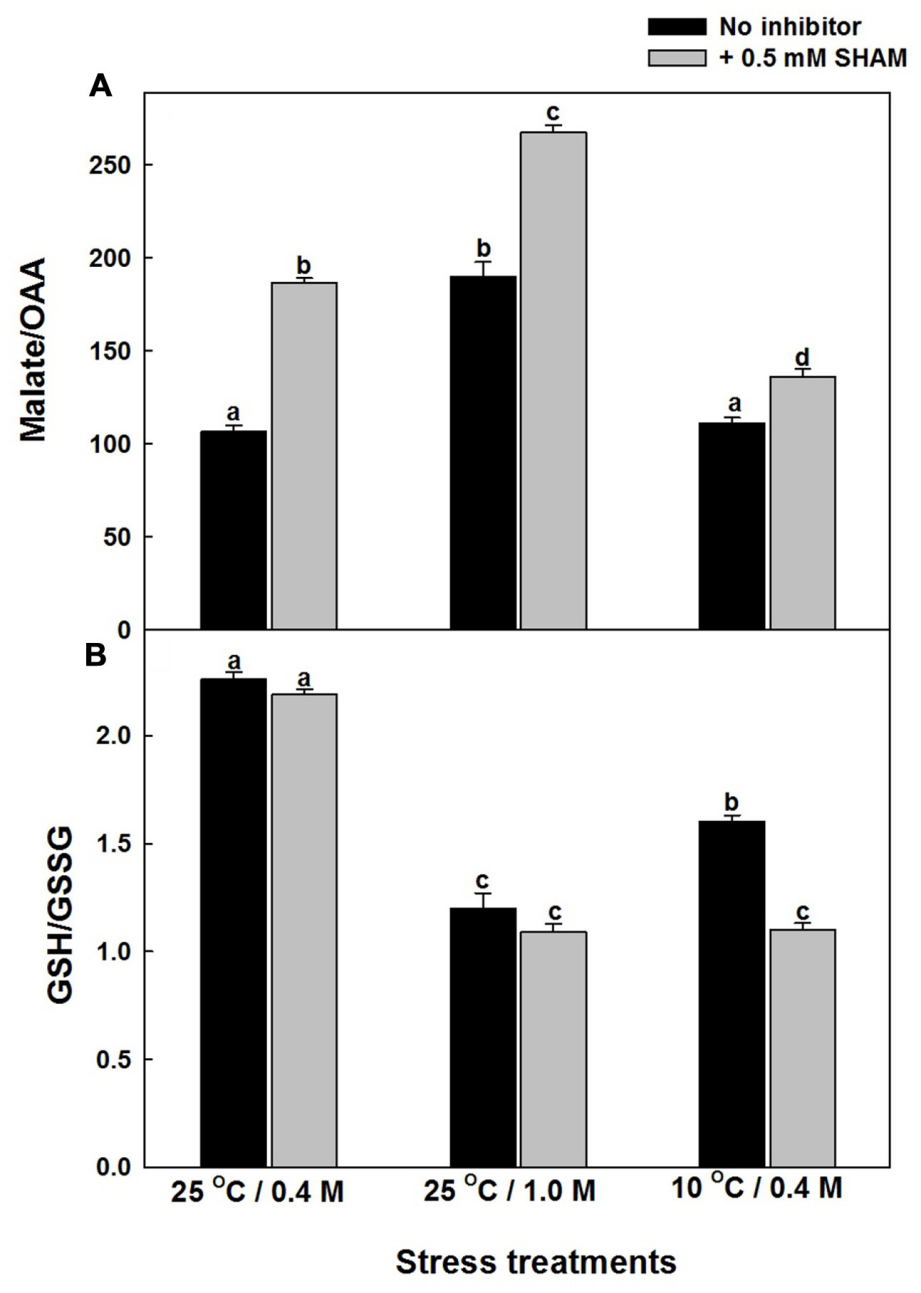
**Effect of 0.5 mM SHAM on malate/OAA **(A)** and GSH/GSSG **(B)** ratio in mesophyll protoplasts pre-incubated under 0.4 M (control), 1.0 M sorbitol (osmotic stress) at 25°C and 0.4 M sorbitol at 10°C (temperature stress), respectively, at a saturating light intensity of 1000 μmol m^–2^ s^–1^ for 10 min.** At the end of the stress treatment, HClO_4_ was added to the reaction medium and the samples were frozen dry in liquid nitrogen for analysis of malate, oxaloacetate as described in section “Materials and methods.” Different letters represent values that are statistically different (ANOVA test, *P* ≤ 0.05).

Any intracellular increase in malate/OAA ratio suggests an imbalance of malate valve, operated to export the photochemically generated reducing equivalents that are in excess of the Calvin cycle requirement (Heineke et al., 1991; Atkin et al., 2000; Scheibe et al., 2005). The malate/OAA ratios of mesophyll protoplasts increased by 79% and 4%, respectively, as compared to control under hyper-osmotic stress or sub-optimal temperature stress and the increase was aggravated significantly upon superimposition with SHAM, under both hyper-osmotic (2.5-fold) and sub-optimal temperature (27%) stresses (Supplementary Figure S2 and **Figure 5A**).

The changes in the redox state of glutathione (an important component of Ascorbate-glutathione cycle) as indicated by the GSH/GSSG levels were decreased by 47% and 30%, respectively, upon treatment with hyper-osmotic stress or sub-optimal temperature stress (Supplementary Figure S3; **Figure 5B**). However, the decrease was more pronounced upon superimposition with SHAM in presence of sub-optimal temperature stress when compared with hyper-osmotic stress (**Figure 5B**). Increase in GSSG levels during stress conditions indicates the oxidation of GSH (Supplementary Figure S3).

**FIGURE 6:**
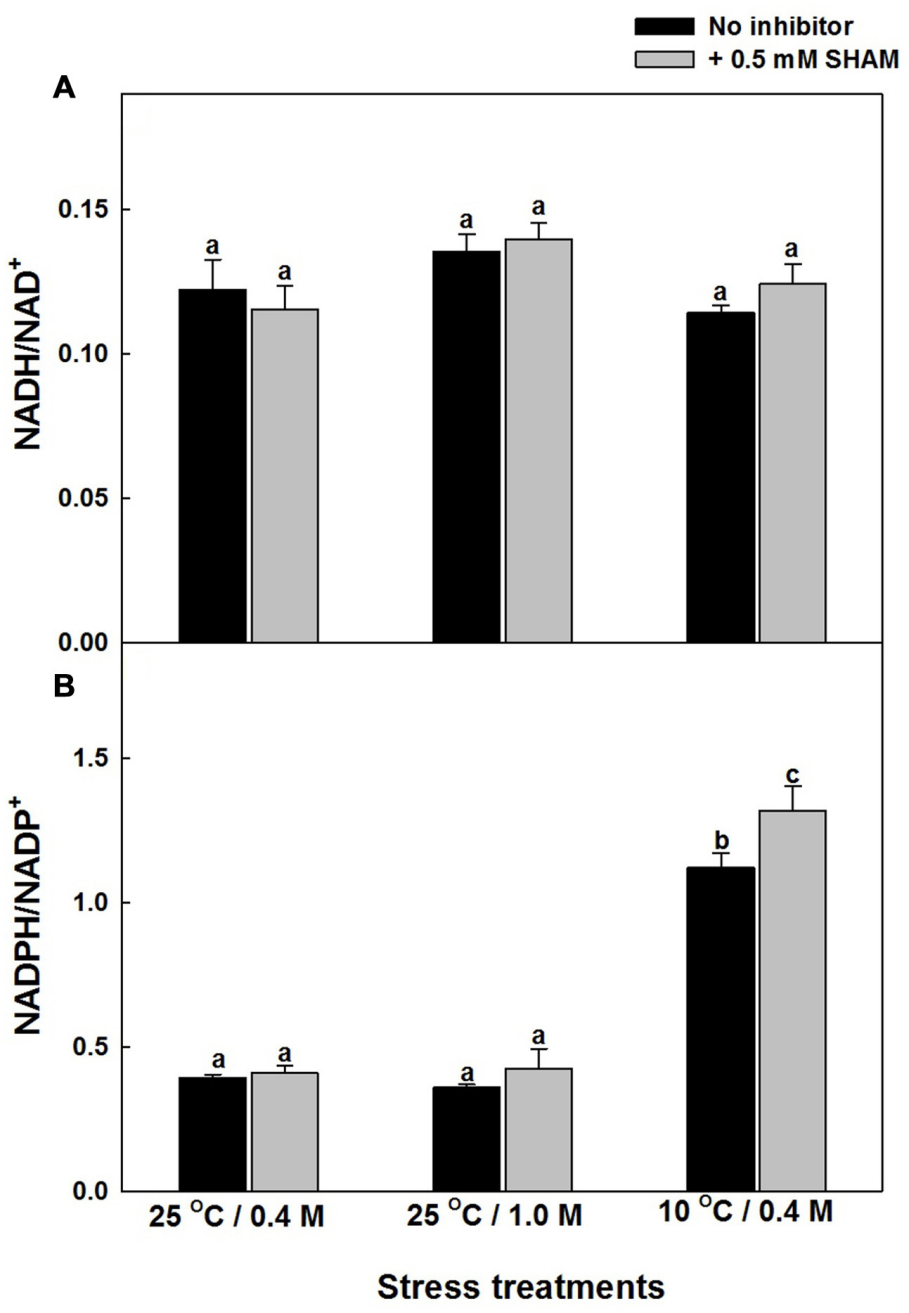
**Effect of SHAM on **(A)** NADH/NAD^+^ and **(B)** NADPH/NADP^+^ ratios in mesophyll protoplasts pre-incubated under 0.4 M (control), 1.0 M sorbitol (osmotic stress) at 25°C and 0.4 M sorbitol at 10°C (temperature stress), respectively, at a saturating light intensity of 1000 μmol m^–2^ s^–1^ for 10 min.** Other details were mentioned in section “Materials and methods.” Different lowercase letters represent values that are statistically different (ANOVA test, *P* ≤ 0.05).

The role of AOX pathway in regulating cellular redox homeostasis during hyper-osmotic and sub-optimal temperature stress conditions was also determined by monitoring the changes in the redox couples related to pyridine nucleotides: NADH/NAD^+^ and NADPH/NADP^+^ in the absence and presence of SHAM (Supplementary Figures S4 and S5; **Figures 6A,B**). Inspite of the significant increase in NADH and NAD^+^, the increase in NADH/NAD^+^ were marginal even after treatment with SHAM under both hyper-osmotic stress or sub-optimal temperature stress (Supplementary Figure S4 and **Figure 6A**). A similar trend in increase of NADPH and NADP^+^ was observed with and without SHAM under hyper-osmotic or sub-optimal temperature stress (Supplementary Figure S5). But, in contrast to redox ratio of NADH/NAD^+^, the redox ratio of NADPH/NADP^+^ increased significantly under sub-optimal temperature stress and was further aggravated upon treatment with SHAM (**Figure 6B**).

### Effect of SHAM on the Activities of NADP-MDH and Antioxidant Enzymes During Hyper-Osmotic Stress and Sub-Optimal Temperature Stress in Light

**FIGURE 7:**
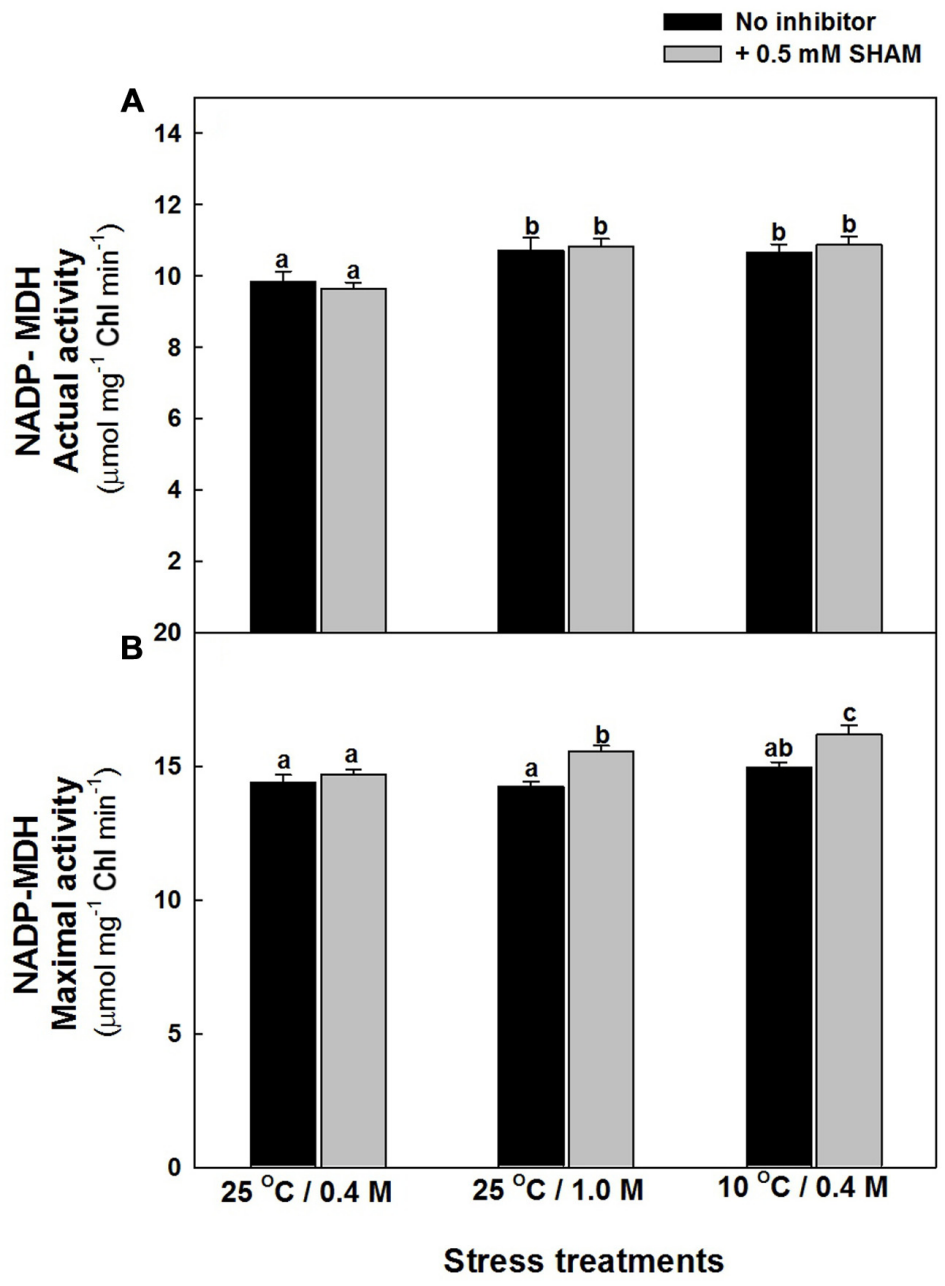
**NADP- MDH actual **(A)** and maximal **(B)** activity in mesophyll protoplasts preincubated under osmotic and temperature stress conditions in the presence and absence of SHAM.** Actual activity was measured directly from supernatant while maximal activity as measured after preincubation for 30 min. Other details were mentioned in section “Materials and methods.” Different lowercase letters represent values that are statistically different (ANOVA test, *P* ≤ 0.05).

The changes in the actual activity of NADP dependent MDH, associated with malate valve was marginal upon treatment of mesophyll protoplasts with hyper-osmotic stress or sub-optimal temperature stress in the absence and presence of SHAM. But, the maximal activity of NADP-MDH was more pronounced upon treatment with SHAM when compared to samples in the absence of SHAM at both hyper-osmotic and sub-optimal temperature stress (**Figures 7A,B**).

**FIGURE 8:**
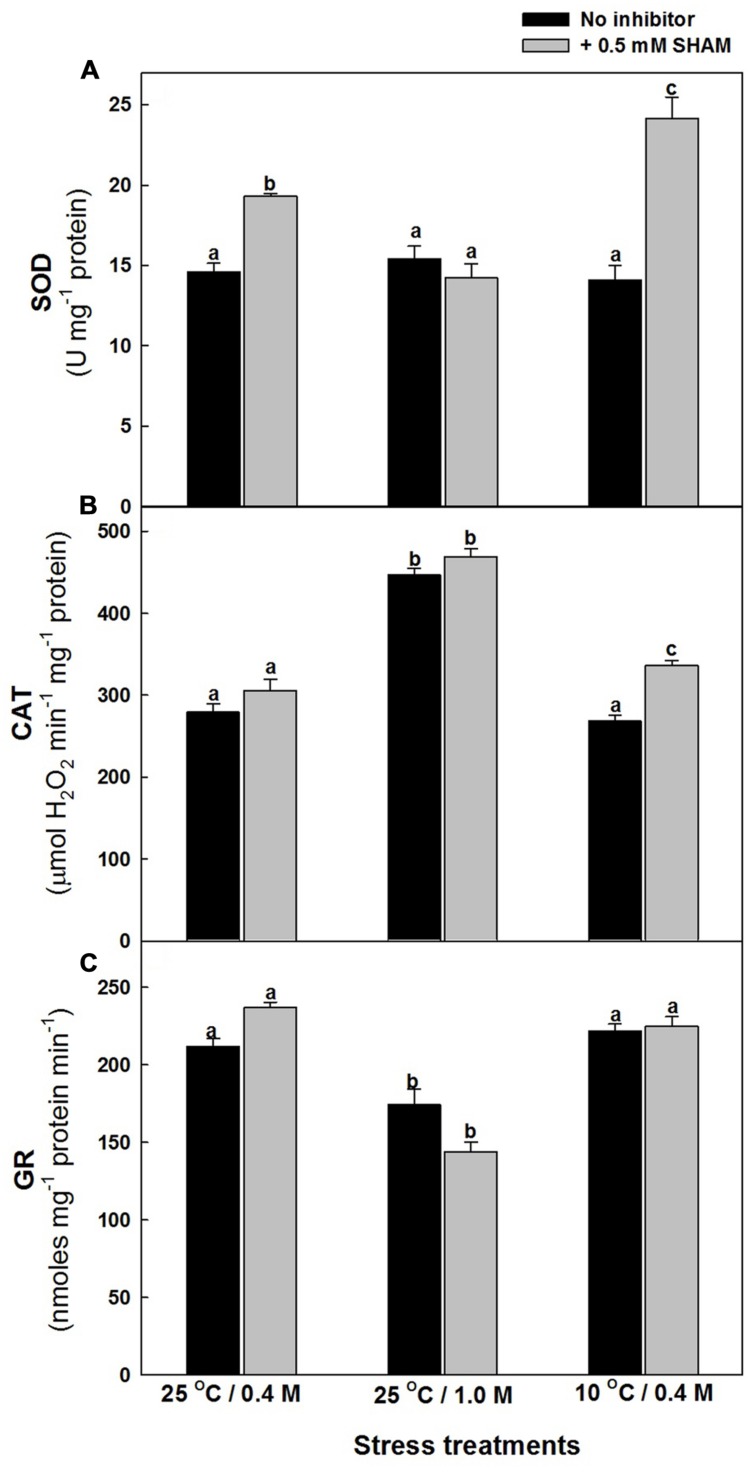
**Effect of SHAM on SOD **(A)**, CAT **(B)**, and GR **(C)** activities in mesophyll protoplasts pre-incubated under osmotic and temperature stress conditions.** Cellular activities of SOD, CAT, and GR were determined by spectrophotometric method in mesophyll protoplasts pre-incubated under 0.4 M (control), 1.0 M sorbitol (osmotic stress) at 25°C and 0.4 M sorbitol at 10°C (temperature stress), respectively, at a saturating light intensity of 1000 μmol m^–2^ s^–1^ for 10 min with and without 0.5 mM SHAM. Different letters represent values that are statistically different (ANOVA test, *P* ≤ 0.05).

The effect of hyper-osmotic and sub-optimal temperature stresses on the activities of antioxidative system, particularly those of ROS generating SOD and ROS scavenging CAT as well as GR, which is involved in ROS scavenging by utilizing redox equivalents were analyzed in the presence and absence of SHAM. The changes in SOD activities were marginal in presence of both stresses examined. However, upon superimposition with SHAM, there was a pronounced increase in the activity of SOD in presence of sub-optimal temperature stress but not under hyper-osmotic stress (**Figure 8A**). Contrary to SOD activity, the activity of CAT increased significantly by 60% as compared to control under hyper-osmotic stress, while the changes were negligible under 10°C temperature. Also, the superimposition of SHAM increased the activity of catalase furthermore under both stresses (**Figure 8B**). The activity of GR decreased under 1.0 M sorbitol, while changes were negligible under 10°C temperature. Nevertheless, the changes were marginal on superimposition with SHAM under both the given stresses (**Figure 8C**).

Taken together, the results from the present study demonstrate that the AOX pathway play a significant role in optimizing photosynthesis by regulating cellular ROS through redox couples related to malate valve, antioxidative system and pyridine nucleotides.

## Discussion

Chloroplasts and mitochondria are the key organelles that are involved in meeting the energy demands and maintaining the redox homeostasis (Griffin and Turnbull, 2012). Therefore, the metabolic interactions between these organelles through cytosol and/or peroxisomes are mutually beneficial to each other and though reported earlier are still being actively investigated under different biotic and environmental cues (Raghavendra et al., 1994; Krömer, 1995; Padmasree et al., 2002; Raghavendra and Padmasree, 2003; Fernie et al., 2004; Noguchi and Yoshida, 2008; Huang et al., 2013; Sunil et al., 2013; Vanlerberghe, 2013; Shaw and Kundu, 2015). Several of these studies indicated that a marginal interference in electron transport through COX or AOX pathways of mitochondrial electron transport chain using metabolic inhibitors and transgenic mutants/reverse genetic approaches caused a significant drop in photosynthetic carbon assimilation at optimal/limiting CO_2_, saturating/sub-saturating/highlight and optimal/sub-optimal growth conditions, thereby signifying the importance of mitochondrial electron transport for optimizing photosynthesis (Krömer et al., 1993; Padmasree and Raghavendra, 1999a; Dutilleul et al., 2003; Yoshida et al., 2006; Dinakar et al., 2010a,b; Araújo et al., 2014). The most recent study on diatoms using metabolic inhibitors AA and SHAM as well as knockouts of AOX also demonstrated that the export of reducing power generated in the plastid to mitochondria and the import of mitochondrial ATP into plastid is mandatory for optimized carbon fixation and their growth (Bailleul et al., 2015).

Mesophyll protoplasts can be used as an excellent model system over whole plants, leaves or leaf discs to study beneficial interactions between chloroplasts and mitochondria for the following reasons: (i) allow free diffusion of O_2_ and CO_2_ which minimizes the artifacts associated with stomatal patchiness, (ii) devoid of intercellular spaces and cell walls, major hurdles for the passage of metabolic inhibitors/activators and (iii) allow usage of metabolic inhibitors at low concentrations and (iv) allow monitoring of metabolic processes quickly (Padmasree and Raghavendra, 1999a,b,c; Strodtkötter et al., 2009; Dinakar et al., 2010a,b). Under the chosen conditions of isolation, the mesophyll protoplasts did not show any damage or loss in integrity of plasma membrane when stored on ice for several hours. The oxygen evolution rates were steady up to 30 min at 25°C and 0.4 M sorbitol, under light intensity of 1000 μmoles m^–2^ s^–1^ (data not shown; Saradadevi and Raghavendra, 1994). However, they tend to lose their stability upon prolonged incubation at room temperature. Further, the light intensity applied to attain maximal rates of photosynthesis is known to vary in mesophyll protoplasts isolated from different leaves (Riazunnisa et al., 2007; Dinakar et al., 2010a,b). Considering these factors, we restricted the study to a total time period of <30 min which include: hyper-osmoticum (or) sub-optimal temperature stress treatment in light for ‘10 min’; followed by a ‘5 min’ respiratory O_2_ uptake in darkness and subsequently ‘10 min’ photosynthetic O_2_ evolution in light, to monitor the effect of stress on respiration and photosynthesis (**Figures 1A–D**). Inspite of the known non-specific effects of SHAM, it is frequently used to assess the role of AOX. It is easily permeable through the plasma membrane and at the concentration (0.5 mM) used in the present study, it neither affected photosynthesis nor ROS in isolated chloroplasts (Padmasree et al., 2002; Dinakar et al., 2010a; Bailleul et al., 2015). While our previous studies emphasized on the importance of COX and AOX pathways in optimizing photosynthesis (Padmasree and Raghavendra, 1999a,b,c, 2001a,b; Strodtkötter et al., 2009; Dinakar et al., 2010a; Vishwakarma et al., 2015) and protecting photosynthesis from photoinhibition under high light (Saradadevi and Raghavendra, 1992; Dinakar et al., 2010b; Vishwakarma et al., 2014), the present study demonstrates the importance of AOX pathway in optimizing photosynthesis under hyper-osmotic and sub-optimal temperature stresses.

### Hyper-Osmoticum and Sub-Optimal Temperature Treatment Caused Marked Reduction in Photosynthetic Carbon Assimilation but not in Total Respiration

The responses of photosynthesis and respiration in mesophyll protoplasts varied when pre-incubated under hyper-osmoticum or sub-optimal temperature stresses. The results indicated that the optimal conditions to achieve maximum photosynthetic performance (carbon assimilation) and respiratory rates in mesophyll protoplasts as indicated by rates of NaHCO_3_-dependent O_2_ evolution and O_2_ uptake, respectively, were found to be at an osmoticum of 0.4 M sorbitol and a temperature of 25°C under a light background of 1000 μmoles m^–2^ s^–1^ (data not shown).

Any deviation from the optimized conditions, i.e., increasing the sorbitol concentration from 0.4 to 1.0 M (or) decreasing the temperature from 25 to 10°C, lead to a significant reduction in photosynthetic carbon assimilation while the changes in dark respiration are minimal (**Figures 1A–D**). Since the plasma membrane of protoplasts was found to be intact after the short-term hyper-osmotic stress and sub-optimal temperature stress treatments (data not shown), the significant decrease in photosynthetic O_2_ evolution is considered as a direct effect of stress on photosynthetic performance. Berkowitz and Gibbs (1983) using the system of isolated chloroplasts showed that hyper-osmotic stress caused inactivation of light activated chloroplastic enzymes like RuBisco and fructose-1,6-bisphatase due to acidification of stroma induced by low osmotic potential. Therefore, the decrease in light activation of the enzymes might be responsible for the decreased photosynthetic O_2_ evolution rates observed in the present study under stress conditions.

In cold sensitive hibiscus plants, cold stress treatment (10°C) caused reduction in the light dependent electron transport reactions thereby causing decreased photosynthesis suggesting the sensitivity of the photosynthetic system to cold temperatures (Parades and Quiles, 2015). In another study Krause et al. (1988) showed the impairment of thylakoid membranes along with the inhibition of PS I and PS II in frost damaged leaves thereby affecting photosynthesis. In mesophyll protoplasts isolated from the non-hardened and cold acclimated plants, differential responses were seen. While photosynthetic CO_2_ assimilation, chlorophyll fluorescence emission and activities of thylakoids were affected in protoplasts isolated from non-hardened plants, in cold acclimated plants the responses were normal. Inhibition of the light activation of light regulated enzymes fructose-1,6-bisphosphatase, sedoheptulose-1,7-bisphosphatase and ribulose-1,5-bisphosphate carboxylase is also one of the reason for decreased photosynthesis during cold stress (Krause et al., 1988). While the effects of hyper-osmoticum and sub-optimal temperatures on photosynthesis are significant, the effects on total respiration are negligible (**Figure 1**)

### Flexibility of Mitochondrial Electron Transport During Osmotic and Temperature Stress Conditions

The flexibility of the mitochondrial electron transport chain to divert electrons from phosphorylating to non-phosphorylating pathways decrease the over reduction of the electron transport chain components and ROS generation. This flexibility in mitochondrial electron transport chain is also observed in the present study during hyper-osmotic stress and sub-optimal temperature stress as evident by a significant increase in the capacity of AOX pathway with a concomitant decrease in the capacity of COX pathway (**Figures 2A,B**). These results corroborated well with the reports of Ribas-Carbo et al. (2005) in soyabean and Dwivedi et al. (2003) in pea, who showed an increase in AOX pathway activity and decrease in COX pathway activity under water and hyper-osmotic stress, respectively. Their results suggested that the increase in AOX pathway activity was due to direct inhibition of the COX pathway activity. Contrary to these results, the COX pathway activity was shown to be increased during water stress in wheat plants, while the leaf discs of *Saxifraga cernua* showed differential responses in COX and AOX pathway activities on exposure to a range of osmotic potentials from 0.0 to 4.0 MPa using sorbitol (Collier and Cummins, 1993; Zagdanska, 1995). The observed variations in the COX and AOX pathway capacity/activities in different studies might be possibly due to variations in the experimental conditions/techniques used to assess them.

Further, the observed decrease in the total cellular ATP/ADP ratios under different stress treatments as compared to controls (**Figure 2C**) corroborated well with the studies of Flexas et al. (2004) and Ribas-Carbo et al. (2005). The studies of Tezara et al. (1999) suggested that the decline in leaf ATP concentration during water stress is an indicator of impaired photophosporylation, which is one of the main factors limiting photosynthesis under water stress. Pyruvate, being a preferential substrate for mitochondrial oxidation is also known to play a significant role in communicating between chloroplasts and mitochondria to activate AOX protein/AOX pathway. The significant increase in the intracellular pyruvate levels under the hyper-osmotic and sub-optimal temperature stresses emphasizes its importance in stimulating the AOX pathway capacity (**Figures 2A,B,D**). In 10°C grown chick pea plants, application of pyruvate on leaves effectively reduced the oxidative stress by activating the AOX pathway (Erdal et al., 2015). Further, any decrease in the COX pathway activity might generate ROS due to over-reduction of the electron transport chain and AOX pathway is very well known to prevent ROS generation (Wagner and Moore, 1997). Thus, the increased ROS during stress conditions might represent the balance of the COX and AOX pathway capacities in light (**Figure 3**). The up regulation of AOX pathway capacity during osmotic and temperature stress conditions signifies the importance of AOX pathway during stress conditions and also highlights its role in decreasing the deleterious effects on not only mitochondrial respiration but also on carbon metabolism (**Figures 1B,D** and **2A,B**). Mitochondria also possess several dissipative systems: rotenone (in)sensitive external and internal NAD(P)H dehydrogenases and complex I, COX pathway, uncoupling proteins (UCP) and potassium channel which may cooperate with AOX to prevent oxidative stress and thereby optimize photosynthetic carbon assimilation. Perhaps, these dissipative systems cannot be ignored in light of the heterogeneity of AOX effects on different components examined in the present study, which were found to be essential for efficient functioning of chloroplastic photosynthesis (Krömer et al., 1988; Igamberdiev et al., 1998; Møller, 2001; Dutilleul et al., 2003; Sweetlove et al., 2006; Yoshida et al., 2006, 2007; Noguchi and Yoshida, 2008). The studies of Trono et al. (2013) demonstrated that the hyperosmotic stress activate a mitochondrial PLA2 which in turn activate UCP and potassium channel to control ROS generation (Laus et al., 2011).

### AOX Pathway Plays an Important Role in Optimizing Photosynthesis Under Hyper-Osmotic and Sub-Optimal Temperature Stress in Light

Studies using metabolic inhibitors or transgenic/reverse genetic approaches indicated that any interference in mitochondrial oxidative electron transport components and TCA cycle causes a significant drop in photosynthetic carbon assimilation along with reduction in the rate of transpiration, stomatal and mesophyll conductance to CO_2_ (Krömer et al., 1993; Padmasree and Raghavendra, 1999a; Dutilleul et al., 2003; Priault et al., 2006; Yoshida et al., 2006, 2011; Dinakar et al., 2010a; Nunes-Nesi et al., 2010; Florez-Sarasa et al., 2011). The low concentration of SHAM (0.5 mM) used in the present study, neither directly affected the reduction in bicarbonate dependent oxygen evolution rates in chloroplasts (Padmasree and Raghavendra, 1999a; Dinakar et al., 2010a) nor affected the photochemical activities of mesophyll protoplasts (Padmasree and Raghavendra, 2001a). SHAM also inhibits all the isoforms of AOX as evident from studies with knockouts of AOX1a *Arabidopsis* plants (Strodtkötter et al., 2009). The results from present study demonstrated that while the effect of 0.5 mM SHAM on respiratory rates and PS II activities were marginal, the decrease in photosynthetic carbon assimilation was significant (**Figure 3**). Since the D1 protein levels were also unchanged in the presence of SHAM under stress conditions, it can be concluded that the marginal interference in AOX pathway under hyper-osmotic stress and sub-optimal temperature stress caused a remarkable decrease in photosynthetic carbon assimilation with marginal effect on photochemical activities, as evident by changes in D1 protein levels (**Figures 3B–D**). Similar observations were also reported by Saradadevi and Raghavendra (1994), where the photosynthetic rates of mesophyll protoplasts decreased to a significant extent on exposure to solutions of increasing osmolarity. The production of ROS by mitochondria was suggested as the critical factor for the induction of AOX (Clifton et al., 2006; Rhoads et al., 2006) and the respiratory capacities of COX and AOX pathways are known to play a significant role in maintenance of cellular ROS at optimal levels to sustain high photosynthetic rates (Dinakar et al., 2010a). In our studies, although we observed a significant increase in ROS, we did not observe the decrease in D1 protein levels under osmotic as well as temperature stress conditions or even in the presence of SHAM (**Figures 3D** and **4**). These results suggest that the changes observed in ROS during hyper- osmotic stress and sub-optimal temperature stress might be involved in signaling function to activate the cellular defense mechanism, perhaps AOX and ROS scavenging antioxidant system (**Figures 2A,B, 4, 5B and 8**).

### Role of Malate Valve and ROS Scavenging Antioxidant System in Stimulating the *In Vivo* Activity of AOX Pathway to Optimize Photosynthesis Under Osmotic and Temperature Stress in Light

Decrease in photosynthesis is a primary effect that is observed during stress conditions. Under these conditions chloroplastic electron transport components accumulate reducing equivalents thereby preventing electron transport. Chloroplasts generated reducing equivalents may be transferred to mitochondria through several metabolite shuttles that operate between the two compartments. Malate and OAA are the two most important metabolites that are involved in redox shuttling between the chloroplasts, mitochondria, and cytosol. Malate/OAA shuttle is believed to be mediated by malate dehydrogenase and in equilibrium with the cellular NADH/NAD^+^ ratio. The assessment of the total cellular NADH and NAD^+^ levels also depends on the other metabolite shuttles and the activity of the mitochondrial oxidative electron transport. Therefore the possibility of change in intracellular malate/OAA ratio without dramatic changes in NADH/NAD^+^ can occur in a cell. The major change in malate/OAA ratio is expected in chloroplasts, while NADH/NAD^+^ ratio is mostly in cytosol. This may be partly due to the consumption of reduced equivalents from malate by other metabolic components such as GSH and/or ascorbate. The pronounced increase in malate levels under hyper-osmotic stress conditions in the presence of SHAM indicates the biochemical role of malate in chloroplast-mitochondrial interactions (Supplementary Figure S3; **Figure 5A**). Biochemically the malate is oxidized to pyruvate via malic enzyme. In isolated mitochondria, malic enzyme activity is correlated with intramitochondrial pyruvate generation and consequent AOX activation (Day et al., 1995). In another study Yoshida et al. (2007) observed an active malic enzyme in AOX1a knockout plants. The COX and AOX pathways were known to play a significant role in oxidizing the malate and regenerating OAA to keep up the chloroplastic electron transport carriers in the oxidized state, which in turn helps to keep the Calvin cycle active for maintaining optimal photosynthesis (Padmasree and Raghavendra, 1999c; Raghavendra and Padmasree, 2003). The pronounced increase in malate/OAA ratio suggests the importance of ‘malate valve’ in mediating the cross talk between chloroplasts and mitochondria to activate AOX pathway under hyper-osmotic stress (**Figure 5A**). Chloroplastic NADP-dependent malate dehydrogenase (NADP-MDH) is the key enzyme controlling the malate valve, which export reducing equivalents indirectly from chloroplasts. The significant increase in maximal NADP-MDH activity in presence of SHAM corroborate well with the increased NADPH and malate levels, and redox ratios of NADPH/NADP^+^ and malate/OAA, respectively, under both hyper-osmotic and temperature stresses (**Figures 5A, 6B**, and **7B**; Supplementary Figure S2A). However, the marginal increase in malate/OAA ratio in the presence of SHAM during sub-optimal temperature stress denotes that a redox modulating factor other than malate might play a role in modulating the ROS to keep up the Calvin cycle activity in chloroplasts.

While the amounts and activities of enzymes involved in ROS scavenging are known to be altered by environmental stresses such as chilling, drought and high salinity (Shao et al., 2008), the reductive detoxification of ROS occurs through the cellular ascorbate and glutathione pools (Smirnoff, 2000; Noctor, 2006). The decrease in photosynthetic carbon assimilation and GSH/GSSG ratio, parallel to a rise in ROS in presence of SHAM under osmotic and temperature stress suggests the role of AOX in optimizing photosynthesis by regulating ROS through glutathione redox couple (**Figures 3–5**). AOX pathway is known to play a significant role in optimizing photosynthesis by keeping up the light activation of chloroplastic enzymes (Padmasree and Raghavendra, 2001b). As these enzymes are regulated by thioredoxin-glutaredoxins, a remarkable decrease in glutathione redox couple at 10°C in presence of SHAM and increase in AOX pathway capacity provide evidence for the physiological role of AOX pathway in keeping up the light activation of chloroplastic enzymes to sustain photosynthesis under sub-optimal temperature stress (**Figures 2B, 3B**, and **5B**). Further, the marginal changes in NADH/NAD^+^ redox couples, in presence of SHAM when superimposed with hyper-osmotic stress and sub-optimal temperature further confirm the tight coupling of AOX pathway with malate/OAA and GSH/GSSG redox couples in regulating cellular ROS to protect photosynthesis from photoinhibiton and sustain photosynthetic performance of mesophyll protoplasts under these stresses (**Figures 2A,B, 3B, 5** and **6**). The increase in the redox ratio of NADPH/NADP^+^ under sub-optimal temperature stress conditions in the presence of SHAM signifies the importance of AOX in oxidizing excess reducing equivalents (**Figure 6B**). Furthermore, though the changes in SOD and catalase activities were significant in the presence of SHAM during sub-optimal temperature stress, they could not play much role in protecting photosynthesis under hyper-osmotic stress by preventing generation/accumulation of cellular ROS (**Figures 8A,B**). On the other hand, the changes in GR were small but not significant under all conditions examined (**Figure 8C**). The significant increase in NADPH/NADP^+^ ratio with concomitant rise in ROS and a decrease in GSH/GSSG ratio while sustaining GR activity in presence of SHAM at 10°C indicated that AOX pathway optimize photosynthesis by regulating antioxidative system at sub-optimal temperature (**Figures 2B**, **4**, **5B**, **6B** and **8C**). These results suggest that non-enzymatic antioxidants play a significant role over enzymatic-oxidants in regulating cellular ROS during optimization of photosynthesis by AOX.

## Conclusion

The present study demonstrates the importance of AOX pathway in optimizing photosynthesis during hyper-osmotic and temperature stress in light. The increased capacity of AOX pathway during both hyper-osmotic and sub-optimal temperature stress was evident by a parallel modulation in various biochemical factors such as pyruvate, ROS and ATP/ADP levels. Studies using mitochondrial AOX pathway inhibitor SHAM demonstrated that under both osmotic and temperature stress, the AOX pathway optimizes photosynthetic carbon assimilation. The results highlight the flexibility of AOX pathway in interacting with different redox couples related to malate valve (malate/OAA) and antioxidative system (GSH/GSSG) to regulate cellular ROS for optimal photosynthetic performance under hyper-osmotic stress and sub-optimal temperature stress. Since the AOX mutants of pea are not available, studies using *Arabidopsis* are required to further understand the underlying molecular mechanisms.

## Author Contributions

Conceived and designed the experiments: CD, KP, and AR. Performed the experiments: CD and AV. Analyzed the data: CD, KP, and AR. Contributed reagents/materials/analysis tools: KP and AR. Wrote the paper: CD and KP

## Conflict of Interest Statement

The authors declare that the research was conducted in the absence of any commercial or financial relationships that could be construed as a potential conflict of interest.
