# Supplementary material for: Alternative Oxidase Pathway Optimizes Photosynthesis During Osmotic and Temperature Stress by Regulating Cellular ROS, Malate Valve and Antioxidative Systems
Source: Front Plant Sci. 2016 Feb 9;7:68. doi: 10.3389/fpls.2016.00068 (PMC4747084; doi:10.3389/fpls.2016.00068)
Supplement: Supplementary file 1 [file Data_Sheet_1.DOC]

**Supplementary Figure Legends**

**Supplementary Fig.1.** Effect of SHAM on (A) ATP and (B) ADP levels in mesophyll protoplasts pre-incubated under 0.4M (control), 1.0 M sorbitol (osmotic stress) and at 10 ^o^C (temperature stress) at a saturating light intensity of 1000 μmol m^−2^ s^−1^ for 10 min. Different letters represent values that are statistically different (ANOVA test, P ≤ 0.05)

**Supplementary Fig. 2.** Effect of 0.5 mM SHAM on intracellular (A) malate and (B) OAA levels in mesophyll protoplasts pre-incubated under 0.4M (control), 1.0 M sorbitol (osmotic stress) ^o^C and at 10 ^o^C (temperature stress), respectively, at a saturating light intensity of 1000 μmol m^−2^ s^−1^ for 10 min. HClO_4_ was added to the reaction medium at the end of stress treatments and the samples were snap frozen in liquid nitrogen for analysis of malate and oxaloacetate. The cellular levels of oxaloacetate were calculated from the equation of [(oxoglutarate) × (aspartate)] / [(glutamate)× (6.61)], based on the equilibrium of glutamate oxaloacetate transaminase (GOT) as described in materials and methods. The intracellular levels of oxoglutarate, aspartate and glutamate in controls without SHAM were 334.1 ± 12.6, 392.2 ± 11.1 and 1233.3 ± 112 nmoles mg^-1^ chl. Other details were described in “Materials and methods”. Different letters represent values that are statistically different (ANOVA test, P ≤ 0.05)

**Supplementary Fig. 3.** Intracellular levels of (A) GSH and (B) GSSG levels in mesophyll protoplasts pre-incubated under 0.4M (control), 1.0 M sorbitol (osmotic stress) at 25 ^o^C and at 10 ^o^C (temperature stress) in the presence and absence of 0.5 mM SHAM. Details about stress treatments and analysis of GSH and GSSG levels are described in Materials and methods. Different letters represent values that are statistically different (ANOVA test, P ≤ 0.05)

**Supplementary Fig. 4.** Changes in total cellular reduced and oxidized pools of (A) NADH and (B) NAD^+^ in mesophyll protoplasts pre-incubated under 0.4 M (control), 1.0 M sorbitol (osmotic stress) at 25 ^o^C and at 10 ^o^C (temperature stress) in the presence and absence of 0.5 mM SHAM. Other details are mentioned in Materials and methods. Different letters represent values that are statistically different (ANOVA test, P ≤ 0.05)

**Supplementary Fig. 5.** Changes in total cellular reduced and oxidized pools (A) NADPH and (B) NADP^+^ in mesophyll protoplasts pre-incubated under 0.4M (control), 1.0 M sorbitol (osmotic stress) and at 10 ^o^C (temperature stress) in the presence and absence of 0.5 mM SHAM. Other details are mentioned in Materials and methods. Different letters represent values that are statistically different (ANOVA test, P ≤ 0.05)
