# Supplementary figures and images for: Alternative Oxidase Pathway Optimizes Photosynthesis During Osmotic and Temperature Stress by Regulating Cellular ROS, Malate Valve and Antioxidative Systems
Source: Front Plant Sci. 2016 Feb 9;7:68. doi: 10.3389/fpls.2016.00068 (PMC4747084; doi:10.3389/fpls.2016.00068)

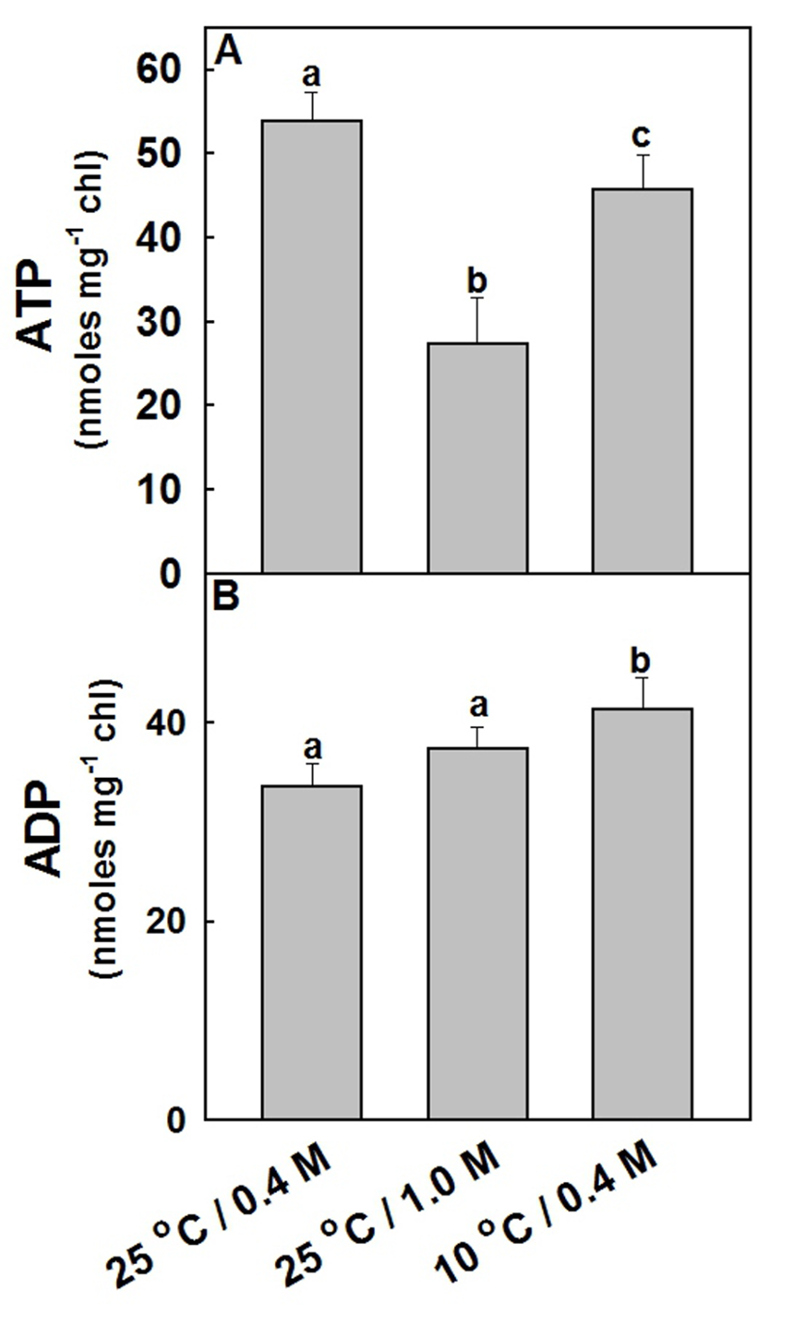

Supplement: Supplementary file 2 [file Image_1.JPEG]

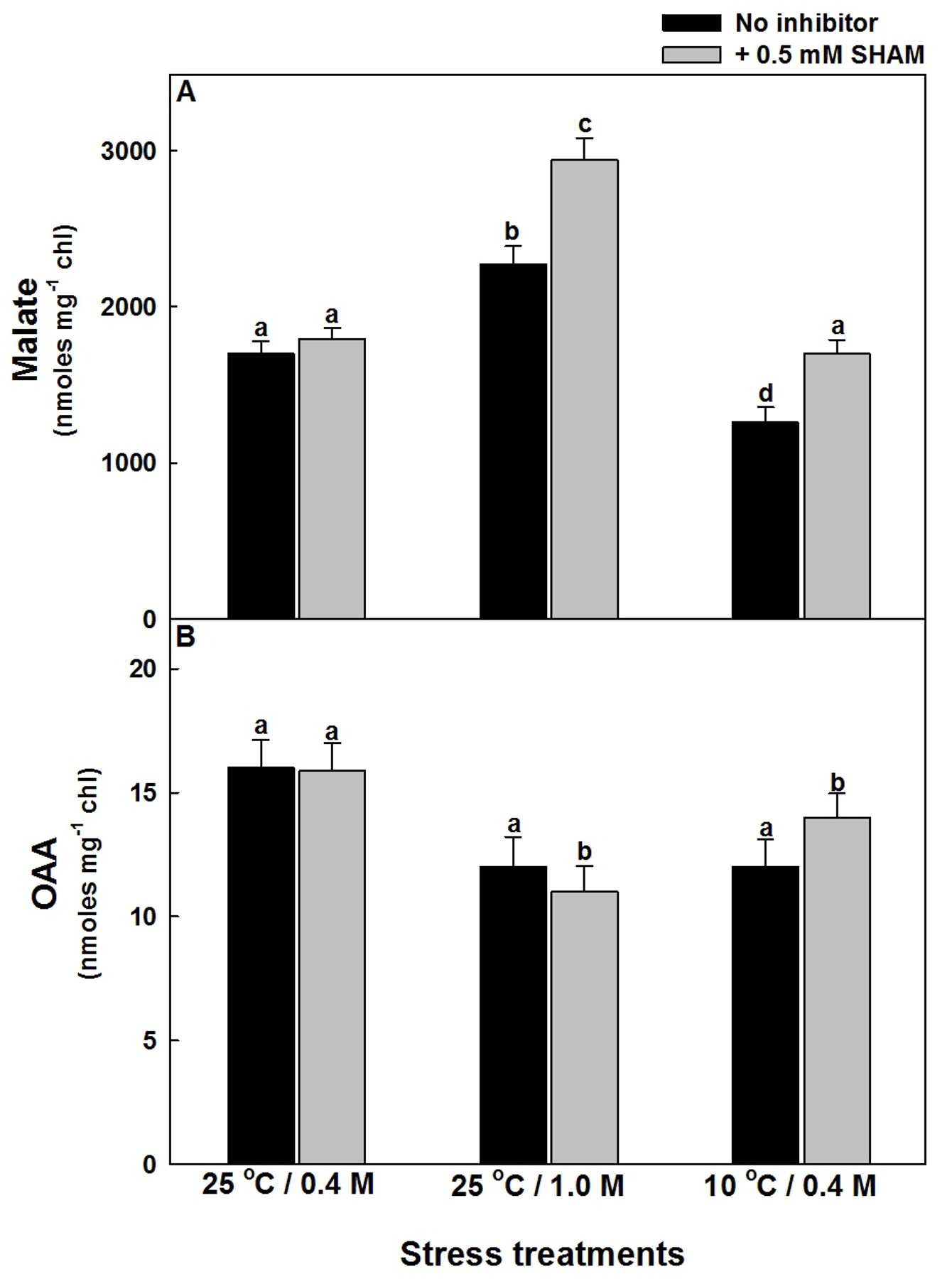

Supplement: Supplementary file 3 [file Image_2.JPEG]

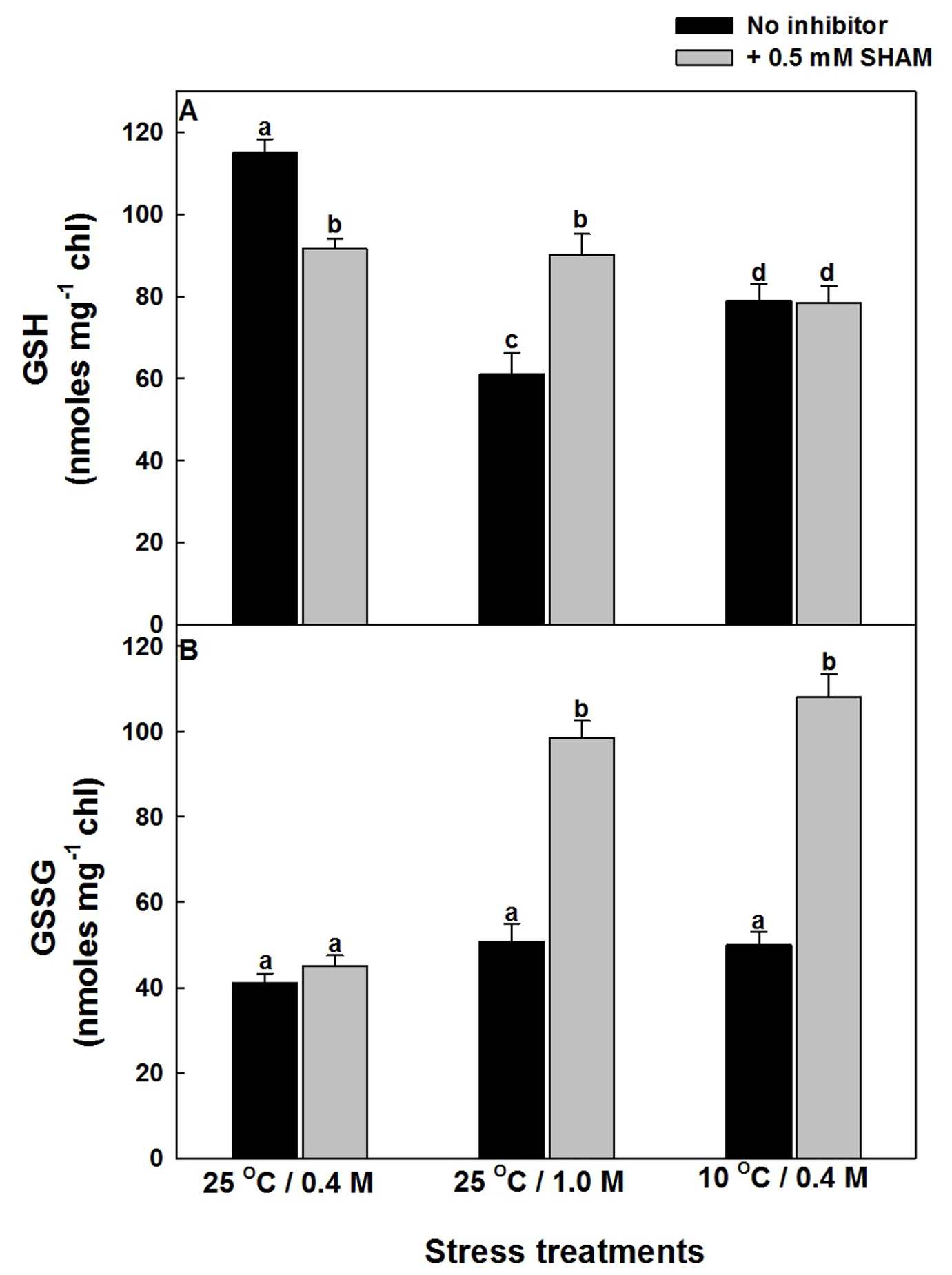

Supplement: Supplementary file 4 [file Image_3.JPEG]

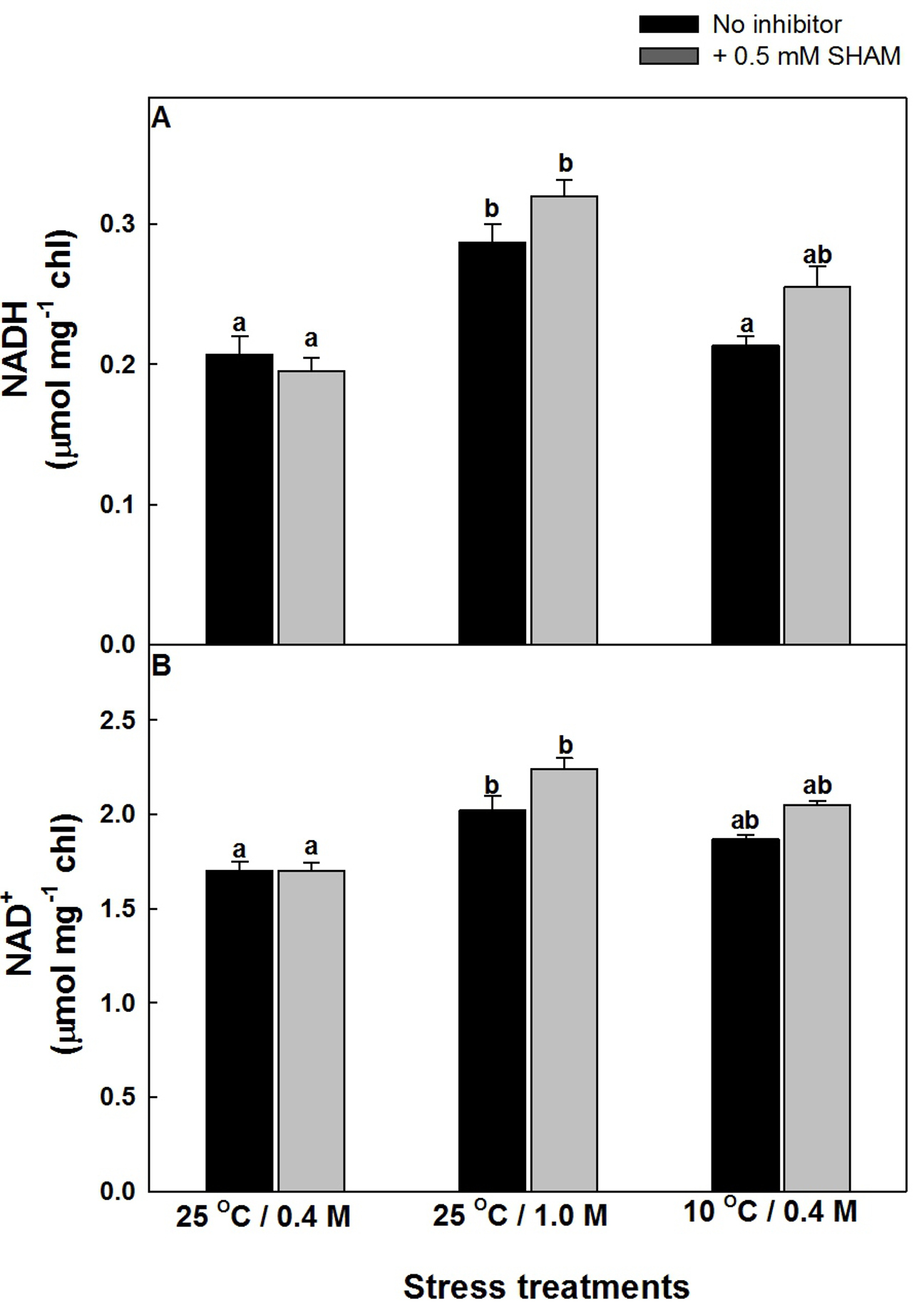

Supplement: Supplementary file 5 [file Image_4.JPEG]

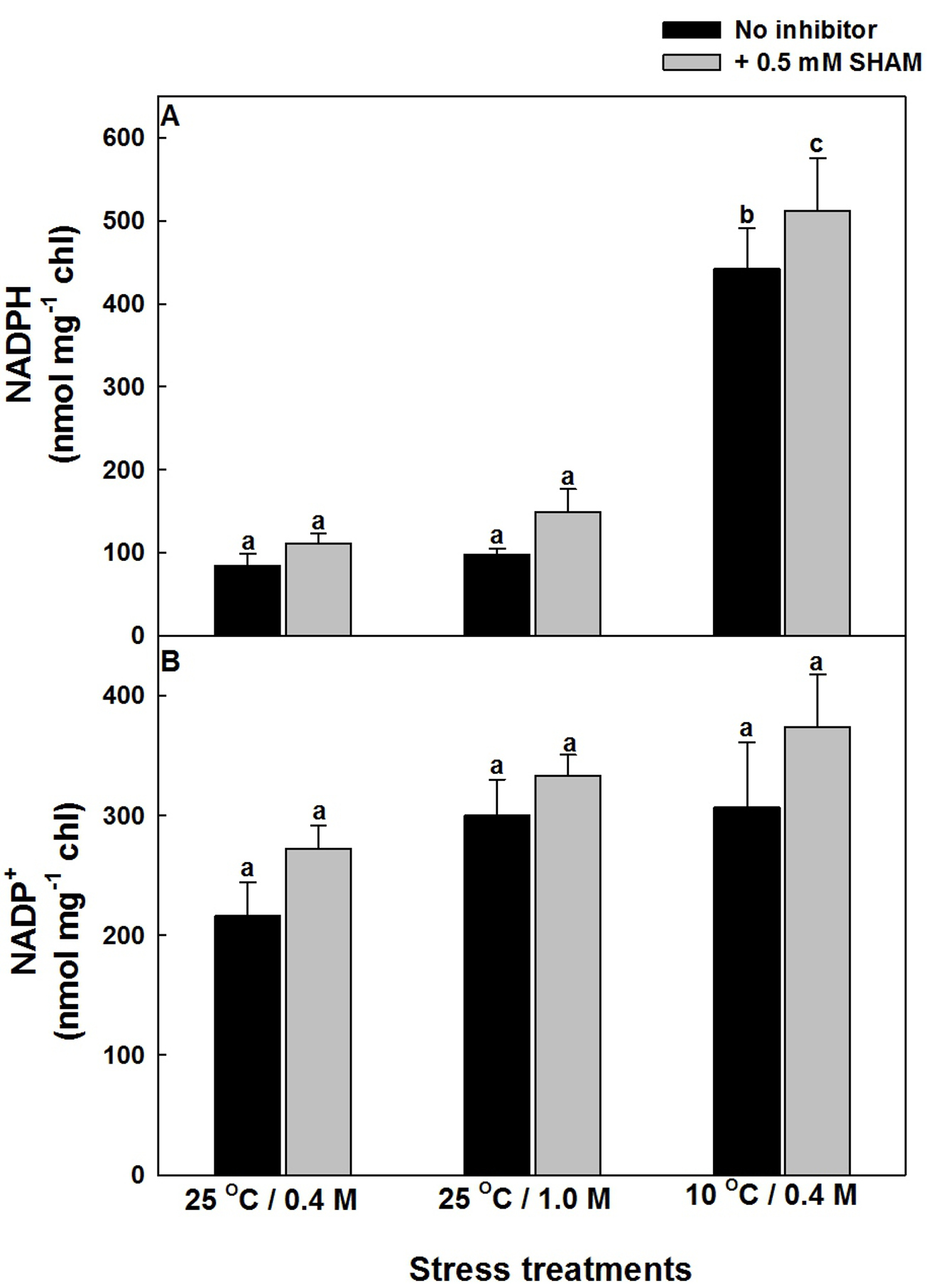

Supplement: Supplementary file 6 [file Image_5.JPEG]
